# Decomposed Dissimilarity Measure for Evaluation of Digital Image Denoising

**DOI:** 10.3390/s23125657

**Published:** 2023-06-16

**Authors:** Łukasz Maliński

**Affiliations:** Department of Industrial Informatics, Silesian University of Technology, Krasińskiego 8, 40-019 Katowice, Poland; lukasz.malinski@polsl.pl

**Keywords:** image denoising evaluation, impulsive noise detection and suppression

## Abstract

A new approach to the evaluation of digital image denoising algorithms is presented. In the proposed method, the mean absolute error (MAE) is decomposed into three components that reflect the different cases of denoising imperfections. Moreover, aim plots are described, which are designed to be a very clear and intuitive form of presentation of the new decomposed measure. Finally, examples of the application of the decomposed MAE and the aim plots in the evaluation of impulsive noise removal algorithms are presented. The decomposed MAE measure is a hybrid of the image dissimilarity measure and detection performance measures. It provides information about sources of errors such as pixel estimation errors, unnecessary altered pixels, or undetected and uncorrected distorted pixels. It measures the impact of these factors on the overall correction performance. The decomposed MAE is suitable for the evaluation of algorithms that perform a detection of the distortion that affects only a certain fraction of the image pixels.

## 1. Introduction

There are numerous image processing methods designed to improve image quality by the removal of different kinds of distortions [1,2,3,4,5,6]. It is obvious that not every distortion can affect the entire image area; thus, every single pixel does not need to be processed. One of the typical problems of such characteristics is image denoising, especially impulsive noise suppression, which has been recently explored and become a popular topic to examine [7,8,9,10,11,12,13]. Other applications, where larger artifacts are detected and removed from the images but not all the pixels are altered, include rain [14,15,16,17,18,19,20], snow [20,21,22,23,24], marine snow [25], and crack [26] removal problems. Furthermore, algorithms that are dedicated to image tamper detection and correction [27] may fit into the above-mentioned description.

Although a plethora of impulsive noise suppression algorithms have been proposed [28,29,30,31,32], a family of switching filters show very promising performance [33,34,35,36,37,38,39], which can only be outperformed by deep learning techniques [39,40]. These switching algorithms separate noise suppression into two steps: impulse detection and impulse correction [39]. In the first step, the pixels are categorized as noisy and noise-free. In the second, only the noisy pixels are replaced by their estimates. Both detection and correction steps acquire information gathered from a local neighborhood of processed pixels. Many of the scalar objective image dissimilarity measures, for example, the PSNR (peak signal-to-noise ratio), the MAE (mean absolute error), the NCD (normalized color distance), and the SSIM/FSIMc (structural/feature similarity) [41], are commonly used for evaluating the image denoising algorithms and many other image processing tasks.

In this paper, a novel vector image dissimilarity measure called decomposed MAE (dMAE) is proposed. It combines the scalar image dissimilarity measure MAE with the detection performance measure such as accuracy, false positive rate, and false negative rate. As a result, it is possible to determine:

- How accurate the correction method used in the evaluated algorithm can be;
- How many errors are accumulated in the undetected noisy pixels, and how much of the collateral distortion is introduced by the evaluated algorithm.

### 1.1. Related Work

A decomposition of the image dissimilarity measure was introduced for gray-scale images by De Angelis et al. [42]. The measures proposed by them were separately computed for noisy pixels (RMSE_A_) and noise-free pixels (RMSE_B_). Further development of the decomposed measure for the color images was completed later by Russo [43]. In his work, the MSE variants (LMSE and CMSE) separated the computation of the denoising errors for luminescence and chromaticity. The author also proposed variants of these measures of computing errors for estimated residual noise (LMSE_a_ and CMSE_a_) and the collateral distortions introduced by the denoising algorithm (LMSE_b_ and CMSE_b_).

The main advantage of the dMAE presented in this paper, over the above-mentioned measures, is the separation of the residual noise into the error caused by the undetected noisy pixels and by the limited accuracy of the estimation of the properly detected pixels. Moreover, the computation of the dMAE does not require additional filtration of the reference image and it is useful for both gray-scale and color images, as well as providing a unique option to perform both pixel-wise and channel-wise analyses.

### 1.2. Structure of the Paper and Author Contribution

The paper is composed of four main sections. In the first section, the introduction to the topic along with the presentation of related work is provided. The second section contains the detailed description of the new dMAE measure. In the third section, examples of use of the dMAE for impulsive noise removal evaluation and algorithm tuning are presented along with a discussion of the results. Finally, the last section contains the conclusions.

The main contributions of this work are:

- The introduction of the dMAE that is considerably simpler to compute than measures proposed in [42,43], yet a very informative hybrid measure of image dissimilarity and algorithm detection capability.
- The proposition of the aim plots, which are the clear and intuitive form of visualization of the dMAE. Furthermore, ready-to-use MATLAB functions [44] were provided.
- A presentation of the examples that use the aim plots and the dMAE in the evaluation of the impulsive noise removal algorithms.

### 1.3. Motivation

The dMAE metric and aim plots presented in the paper were invented during the work on algorithms dedicated to impulsive noise removal. During many tuning approaches, it has been found that tuning algorithms to maximize detection accuracy rarely result in the best overall denoising performance. It was found that the unnecessary correction of noise-free pixels (false-positives) introduces less error than the omission of hard-to-detect noisy ones (false-negatives). It led to the idea of the design of the decomposed measure that provides clear information on which type of error is more damaging to the image.

The aim of the paper is to introduce the concept of decomposed Mean Absolute Error as a new type of measure, which can be used for tuning and evaluation of denoising algorithms. It is important to state that this measure is suitable only if noise or other distortions affect only a certain fraction of the image pixels. The best example of such noise is impulsive noise, but this measure should also be useful for other problems such as snow/rain removal and crack removal. It is, however, useless for the evaluation of the suppression of gaussian noise because this type of distortion affects every pixel in the image. Moreover, algorithms dedicated to removing gaussian noise try to correct every pixel in the image; thus, the entire decomposition concept is useless in such an application.

## 2. Materials and Methods

Three types of images can be distinguished in a typical evaluation of a denoising algorithm:

- Distorted image (d), which is the input of a denoising algorithm. This image contains noise, but it may not necessarily affect every pixel in the image.
- Corrected image (c), which is the output of a denoising algorithm. It contains suppressed noise, but not every pixel has to be altered during the denoising process.
- Reference image (r), which represents the desired (perfect) outcome of the denoising algorithm. Typically, it is an original image, upon which the distorted image was artificially obtained for algorithm evaluation.

**Figure 1:**
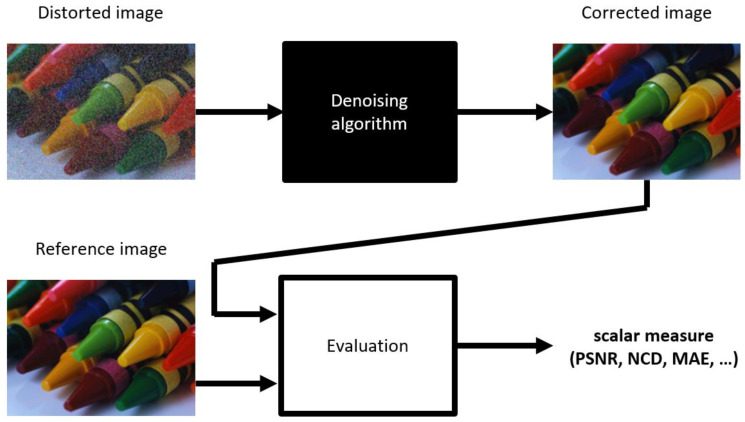
Typical algorithm evaluation scheme.

To rate how much the output of the evaluated algorithm differs from the desired result, one or more dissimilarity measures are computed using the corrected and reference images (Figure 1). Different dissimilarity measures offer various criteria for the evaluation, and their optimization is a typical approach for tuning the performance of the denoising algorithms.

Regardless of the scalar dissimilarity measure used, this type of single-value evaluation can only show that one result is objectively better or worse than the other, within the given criteria. It does not provide any further insight into what the probable source of the difference is. It is important because the three main sources of image denoising imperfections can be distinguished via:

- Inexact estimates of the noisy pixel’s replacements, which are mostly dependent on the accuracy of the estimated pixel’s colors that is obtained during the correction step;
- Non-suppressed noisy pixels, which occur solely due to the imperfection of the detection step.

**Figure 2:**
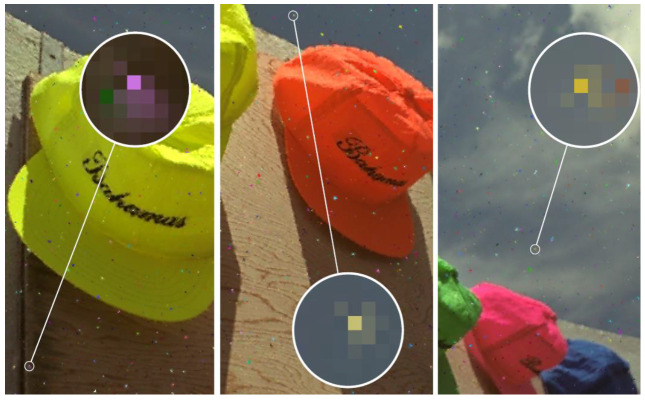
Phantom effect caused by undetected impulses (taken from [45]).

Unnecessarily altered noise-free pixels are mainly caused by detection errors, but they are also dependent on the correction performance. Moreover, for many of the correction algorithms, the undetected impulses introduce additional errors in estimates of the neighboring pixels, which is further referenced as the phantom effect. These undetected impulses are recognized as noise-free pixels by the correction algorithm. If their neighboring pixels are considered to be noisy, and are being corrected, the undetected impulses are taken as “valid” sources of information, while their colors are mostly very distinct from the original pixels that they replace. As a result, the color of the undetected impulses tends to contaminate neighboring pixels, which are properly detected and corrected (Figure 2). Therefore, errors originating from sources 1 and 3 (in the above list) can be amplified by this phantom effect.

The ability to measure the impact of different types of errors on denoising performance is a significant factor, which should be accounted for during the algorithm design and parametrization. Thus, the dissimilarity measure, which can distinguish the impact of the particular sources of errors on the denoising performance, becomes a natural step toward the development in this field of expertise.

As stated before, it is likely that not every pixel in the image is noisy; this is why it should not be affected by the denoising algorithm. Moreover, not every denoising algorithm alters every single pixel in the image, or the result of the processing does not change the color of some pixels at all. These observations lead to the idea that the image dissimilarity measure can be decomposed into three components:

- True-positive dissimilarity (TPD)—computed only for the pixels that should be corrected and have been processed by the denoising algorithms. This component is most suitable to evaluate the actual performance of the noise correction part of the algorithm.
- False-negative dissimilarity (FND)—computed for pixels that should be corrected (i.e., when noisy), yet have not been processed, or have not been changed during the processing. This component reflects how difficult it is for the algorithm to correctly detect impulses.
- False-positive dissimilarity (FPD)—computed for pixels that are unnecessarily altered because they are noise-free. This component rates how much collateral distortion has been introduced by image processing.

True-negative dissimilarity—a possible fourth component, which is not relevant, because these pixels should be identical in the corrected and the reference image and they do not introduce any errors.

There are two ways to obtain the decomposed dissimilarity: *indirect* and *direct*. The indirect way requires the corrected and reference images and two binary maps of distortion (noise):

- True map (TM)—provides true information on which pixels are noisy (marked as 0) and which ones are not (marked as 1);
- Detection map (DM)—provides information on which pixels are classified as noisy (when processed by the denoising algorithm—marked as 0), and which are not (marked as 1).

**Figure 3:**
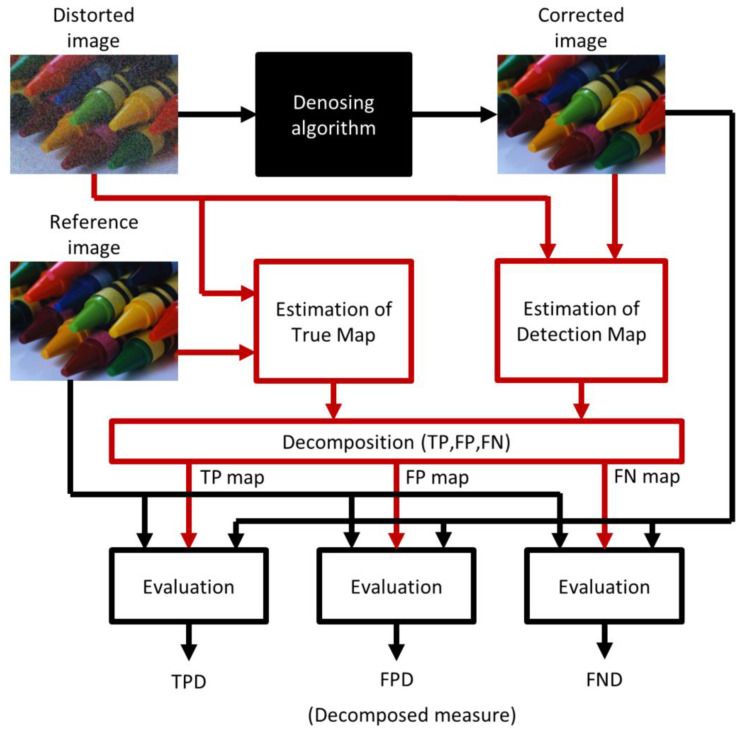
Proposed decomposed evaluation scheme.

In a direct way (Figure 3), all three images: distorted, corrected, and reference, are required, and the maps (TM/DM) are estimated in the process. The comparison of the distorted and reference images provides information about the pixels/channels that should be corrected (1). The comparison of distorted and corrected images provides knowledge of which pixels/channels are altered by the denoising algorithm (2).

(1) $$T_{i,j,h}=\left\{\begin{array}{ll} 1, & \left|d_{i,j,h}-r_{i,j,h}\right|=0,\;\;\\ 0, & \;\;otherwise, \end{array}\right.\;$$

(2) $$D_{i,j,h}=\left\{\begin{array}{ll} 1, & \left|d_{i,j,h}-c_{i,j,h}\right|=0,\\ 0, & \;\;otherwise, \end{array}\;\right.$$

where *i* and *j* are the pixel’s coordinates, *h* is the pixel’s channel (*h* is always 1 for grayscale images or it takes values from 1 to 3 for RGB images), *T_i,j,h_* is a channel class in the true map, *D_i,j,h_* is a channel class in the detection map, *d_i,j,h_* is a pixel channel value in the distorted image, *r_i,h,j_* is the pixel channel value in the reference image, and *c_i,j,h_* is a pixel channel value in the corrected image. For RGB images, both maps are initially made in a channel-wise (CW) manner, which means that each channel of the image is classified separately. Then, these are optionally reduced to pixel-wise (PW) versions, using simple rules:

- If at least one channel of the pixel is classified as distorted, the entire pixel is classified as distorted (true map).
- If at least one channel of the image is altered by the processing algorithm, the entire pixel is classified as altered (detection map).

This is the simpler and the most universal method, but slightly less accurate because the estimated DM might contain some errors. There are algorithms that may not alter some of the pixels during the processing. In the estimated DM, these pixels are recognized incorrectly as the false-negatives. Therefore, the direct way is the most suitable if the evaluated algorithms do not provide access to the DM, or even when they do not perform detection at all.

When both maps, the TM and DM, are acquired or estimated for every pixel, a category *Q* (true positive, false positive, false negative, or true negative) is assigned, so that:

(3) $$Q_{i,j}=\left\{\begin{array}{l} \begin{array}{ll} \mathrm{TP}, & T_{i,j}=0\;and\;D_{i,j}=0,\\ \mathrm{FP}, & T_{i,j}=1\;and\;D_{i,j}=0, \end{array}\\ \begin{array}{ll} \mathrm{FN}, & T_{i,j}=0\;and\;D_{i,j}=1,\\ \mathrm{TN}, & T_{i,j}=1\;and\;D_{i,j}=1, \end{array} \end{array}\;\right.$$

### 2.1. Decomposed Dissimilarity Measure

Among the many candidates, the image dissimilarity measure that is most suitable for the decomposition and presentation is the MAE, because it fulfils the following requirements:

- A perfect match of the two images is reflected in the zero value of the measure.
- The measure is linear. Thus, after decomposition, the sum of the components is equal to its scalar value.

The decomposed MAE (4) is computed as follows:

(4) $${\mathrm{MAE}}_{Q}=\frac{1}{\left|Q\right|H}\sum_{C}\underset{Q}{\sum }\left(\left|y_{i,j,h}-{\hat{y}}_{i,j,h}\right|\right)$$

where *Q* is the category of a pixel (TP, FP, and FN), |*Q*| is the cardinality of category *Q*, *h* is the channel of the image, *H* is number of channels in the image (1 or 3), and *i* and *j* are pixel spatial coordinates, while *y* denotes a pixel in the reference image and $\hat{y}\;$ is a pixel in the restored image.

The proposed measure, like many others (PSNR, MAE, MSE, NCD, SSIM/FSIM, etc.), requires the availability of a reference image that is the equivalent of perfect processing. This means that it is most suitable for designing and tuning the algorithms with artificially corrupted images. This measure is not intended to be used to assess the processing of naturally corrupted images unless a reference image is somehow available.

### 2.2. Form of Presentation

**Figure 4:**
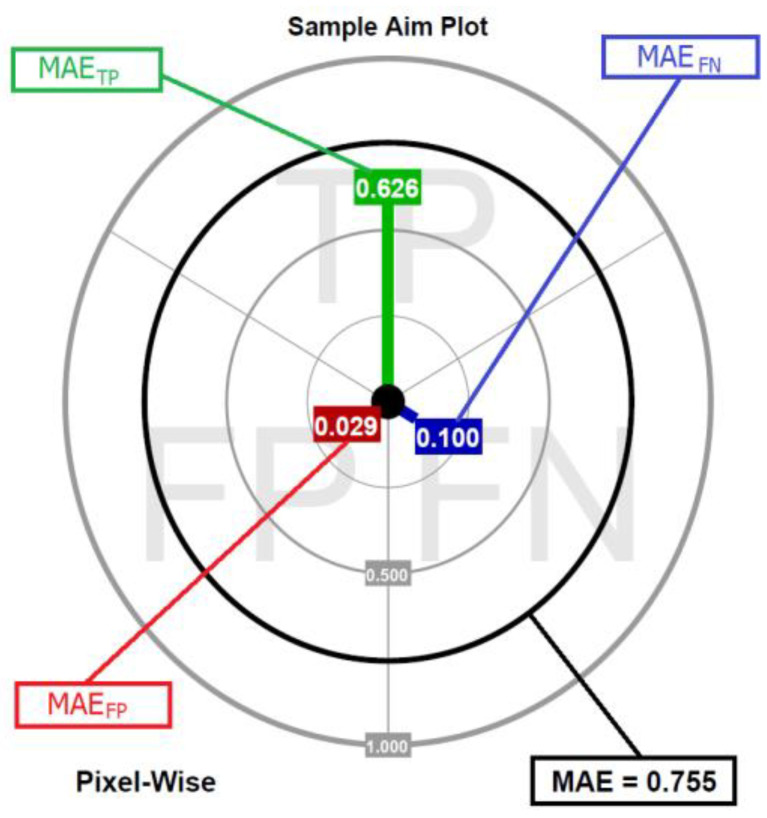
Aim plot showing the decomposed MAE.

To make the presentation of the dMAE clear and intuitive, aim plots are proposed and an example is shown in Figure 4. The colored lines represent the particular components of the dMAE, while the black circle is the scalar value of the MAE (the sum of all three components).

The aim plot might be interpreted as a metaphorical dart board. The “player” (evaluated algorithm) has three darts (green for the MAE_TP_, blue for the MAE_FN_, and red for the MAE_FP_) and “throws with them” toward the three sections of the board. In the perfect scenario, the “player” hits the center of the board three times. However, in real scenarios, there is always a displacement caused by the sources of imperfections depicted in the previous section. The further the distance from the center, the higher the contribution of the dissimilarity error of a particular type. With the ready-to-use Matlab function, aim plots were prepared and published [44].

### 2.3. Type of Analysis

Considering the perspective of both the image processing and performance evaluation, if the processed image is composed of multiple channels (such as RGB digital color images), two types of analysis might be distinguished:

- Pixel-wise (PW)—the pixels are treated as composites. Therefore, during the processing, if at least a single channel of a pixel is distorted, the entire pixel is corrected on every channel. In this evaluation, all errors that occur in channels of such pixels are assigned to the same component of the decomposed dissimilarity, which is based on pixel classification.
- Channel-wise (CW)—in this processing, the distortion on each channel is processed independently; thus, each channel of the same pixel might be assigned to a different component of the decomposed dissimilarity.

## 3. Results and Discussion

Impulsive noise suppression is the most suitable for the presentation of the decomposed MAE measure and the aim plots. In this case, only a certain fraction of image pixels is affected by noise by replacing the original pixels with random colors. If the pixel becomes noisy and a random combination of its channels is altered, the channel-independent random impulse (CIRI) noise model is recognized. When all the pixel channels are changed, the channel together random impulse (CTRI) noise model is identified [46].

**Figure 5:**
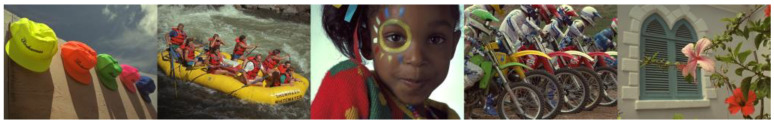
Benchmark image dataset (taken from [45]).

The presentation of the use of the decomposed MAE is divided into four experiments. In the first one, the performance of a set of denoising algorithms was evaluated using the PSNR, the scalar MAE, and the decomposed MAE on images artificially distorted by 20% of the CTRI noise. The second experiment is the application of the decomposed MAE for analysis of the tuning process of the denoising algorithm. In the third, the benefit of using the CW analysis for processing images that are distorted by the CIRI noise is shown. Finally, the fourth experiment shows the statistical properties of dMAE and its comparison to commonly used detection performance measures. For the needs of the experiments presented in this section, a dataset of 5 color images is prepared (Figure 5), which contains selected samples from [45].

### 3.1. Multiple Denoising Algorithms’ Evaluation (Experiment I)

The set of denoising algorithms chosen for the presentation of the proposed evaluation method contains:

- The vector median filter (VMF) [39];
- The fast peer-group detector with the arithmetic mean filter corrector (FPG-AMF) [39];
- The fast adaptive trimmed switching detector with the arithmetic mean filter corrector (FAST-AMF) [39];
- The fast adaptive trimmed switching detector with the inpaint nans corrector (FAST-IPN) [39,47].

The VMF is the most basic, non-switching algorithm used for impulsive noise suppression. It is chosen to show that the decomposed MAE is also suitable for the evaluation of the algorithms that do not perform noise detection at all. The next two modular switching algorithms, the FPG-AMF and FAST-AMF, have a common corrector, but these two use different detectors. They are chosen to show how the decomposed MAE measures the differences in detection performance. Finally, the FAST-IPN has a common detector with the FAST-AMF, but a different corrector. This enables the possibility of comparing the differences in the correction part, while detection performance remains identical. All the detectors use default values for their parameters. Moreover, as the VMF is taken for the evaluation, all the comparisons are made using the direct version to ensure the same computation method. The indirect version might still be used in this comparison if the TM is available (DM is an array full of zeros, which means that every pixel is treated as noisy).

The first comparison is made using scalar measures, such as the PSNR (the higher value is better) and the MAE (the lower value is better). The results are presented in Table 1 and Table 2.

It can be stated that all considered switching filters perform much better than the VMF, and the FAST detector is improved compared to the FPG detector, as well as the IPN corrector being superior to the AMF corrector. These remarks might only apply to a very specific design of the experiment, and the scalar measures do not provide much more insightful information. Furthermore, it is worth mentioning that different ranges of measured values can be obtained for various images; this depends on the image complexity. Images no. 1 and no. 4, which contain the best and worst values of measures, respectively, are obtained and are further referenced as the easiest and the hardest image, respectively.

**Figure 6:**
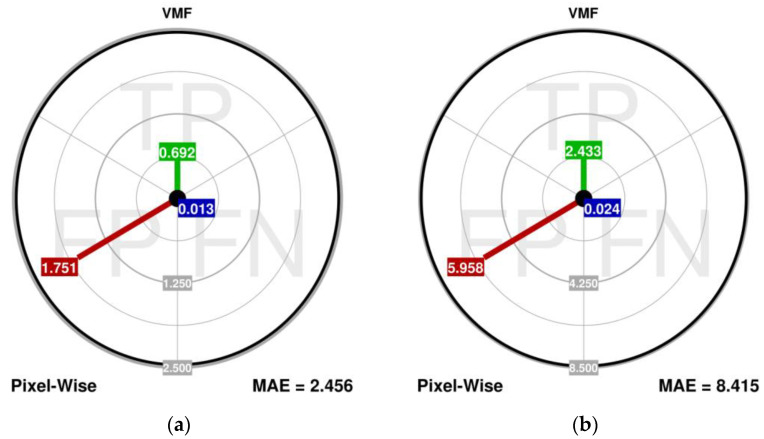

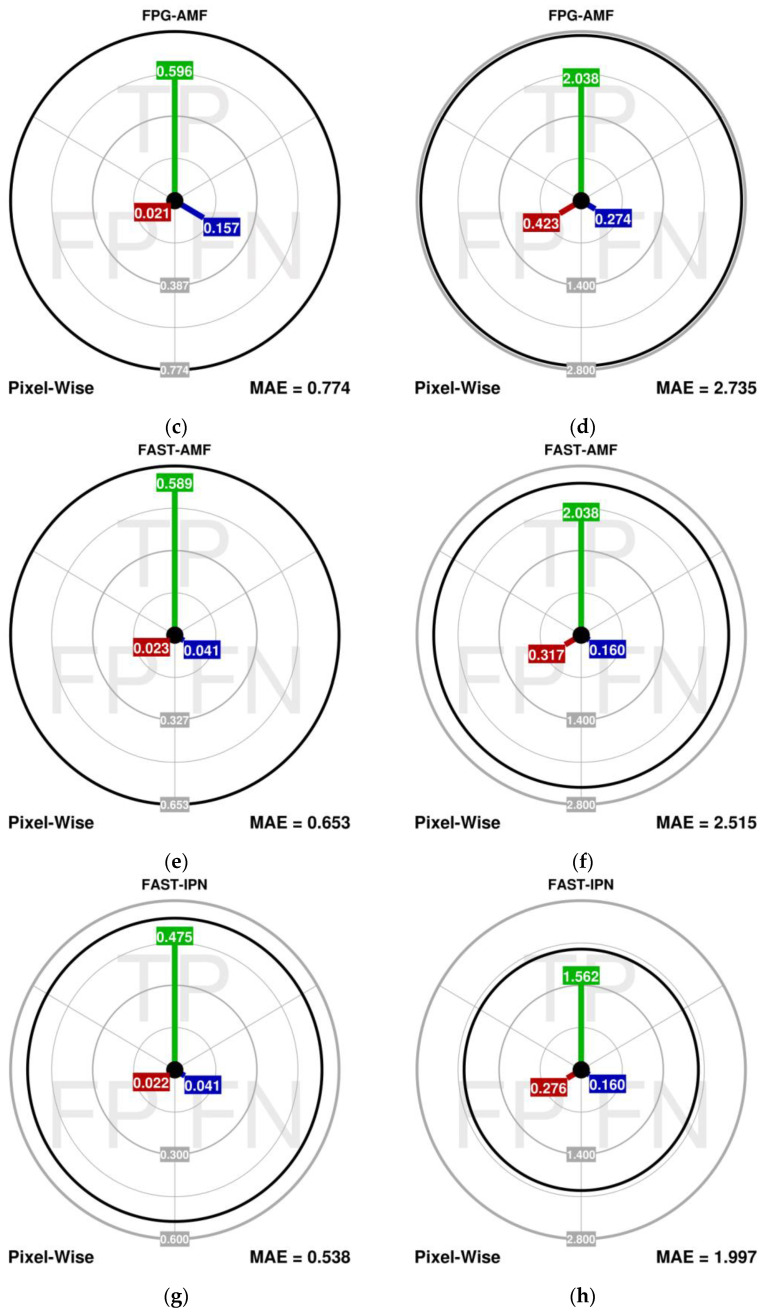
Aim plots for the evaluation of the algorithms (the scale is unified for switching algorithms, which is only possible due to significantly improved performance in comparison to the VMF) for: easiest (**a**,**c**,**e**,**g**) and hardest (**b**,**d**,**f**,**h**) images.

The dMAE, for which the lower values are superior, is obtained for every image and the results are presented in Table 3. The aim plots for the easiest and hardest images are shown in Figure 6 and these should be analyzed in rows. The conclusions that can be drawn from using the scalar measures only are clearly reflected in the dMAE values and the aim plots. However, it can additionally be stated that:

- The dMAE and aim plots show that the most dominant component of the error, made by the VMF, is reflected in the MAE_FP_. It is the result with the VMF lacking noise detection and performing a “correction” on every pixel in the image.
- The VMF shows the worst correction performance in the MAE_TP_. However, surprisingly, a low value of the MAE_FN_ can be noticed for the VMF, which is caused by the estimation of the TM and DM from the images. Even though the VMF processes every pixel of the image, some noisy pixels are not altered.
- The FAST detector has improved impulse detection capabilities, especially in the MAE_FN_ component. It shows that the impact of the omitted noisy pixels is also far less damaging.
- The FAST detector introduces slightly lower MAE_FP_ values, which is the result of a considerably better capability of the detection on both the FP and the FN sides; the fewer FN results that occur, the less phantom effects that arise.
- The IPN corrector is slightly better than the AMF, which is reflected in the MAE_TP_ and MAE_FP_. Therefore, it provides more accurate estimates of the pixels, and introduces less damage to the falsely detected ones.
- The proportions between the MAE_FP_ and MAE_FN_ depend on the image complexity.

### 3.2. Tuning of the FAST-IPN Algorithm Using dMAE and Aim Plots (Experiment II)

In the second experiment, the FAST-IPN is tuned twice: once using the Mathews correlation coefficient (MCC) (5) and once with the PSNR as a cost function. The MCC is a detection performance measure; thus, the first tuning aims to maximize the overall detection capability. Along with its other common detection measures: the false-positive rate—FPR (6) and the false-negative rate—FNR (7) are used to evaluate the detection performance. The corresponding expressions are:

(5) $$\mathrm{MCC}=\frac{\left|\mathrm{TP}\right|\cdot \left|\mathrm{TN}\right|-\left|\mathrm{FP}\right|\cdot \left|\mathrm{FN}\right|}{\sqrt{\left(\left|\mathrm{TP}\right|+\left|\mathrm{FP}\right|\right)\cdot \left(\left|\mathrm{TP}\right|+\left|\mathrm{FN}\right|\right)\cdot \left(\left|\mathrm{TN}\right|+\left|\mathrm{FP}\right|\right)\cdot \left(\left|\mathrm{TN}\right|+\left|\mathrm{FN}\right|\right)}}$$

(6) $$\mathrm{FPR}=\frac{\left|\mathrm{FP}\right|}{\left|\mathrm{TN}\right|+\left|\mathrm{FP}\right|}$$

(7) $$\mathrm{FNR}=\frac{\left|\mathrm{FN}\right|}{\left|\mathrm{TP}\right|+\left|\mathrm{FN}\right|}$$

where |TP|, |TN|, |FP|, and |FN| are cardinalities of the particular classes of pixels.

**Figure 7:**
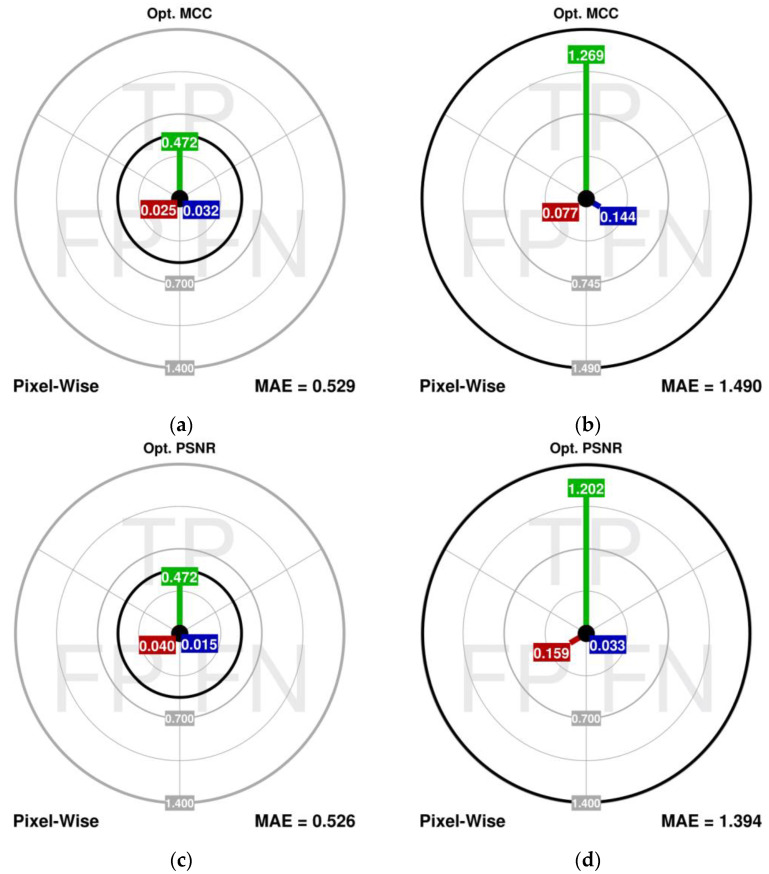
Aim plots for different FAST-IPN tuning scenarios for 20% (**a,c**) and 40% (**b**,**d**) CTRI noise fraction.

The experiment is conducted for image no. 1 distorted with the CTRI noise of fractions 20% and 40%. The comparisons of the scalar measures for the detection and correction are presented in Table 4, while the dMAE is shown in Figure 7.

The results can be summarized as follows:

- Optimization of the detection performance does not translate into the best performance from the image dissimilarity measures’ perspective. The reason behind this is that the MCC operates on raw cardinalities of the detection errors only and takes no account of the actual color differences, which have a major impact on the image dissimilarity measures.
- The higher values of the MAE_FN_ presented in the aim plots are also reflected in the higher values of the MAE_TP_ (for 40% noise fraction), which means that in the impulsive noise suppression, it is the FN errors that can be more harmful than the FP errors (the phantom effect).
- The dMAE is a better measure than the FPR and FNR because it also accounts for the actual impact of these noisy and restored pixels’ color differences on the output image quality.

### 3.3. Difference between the PW and CW Analysis (Experiment III)

**Figure 8:**
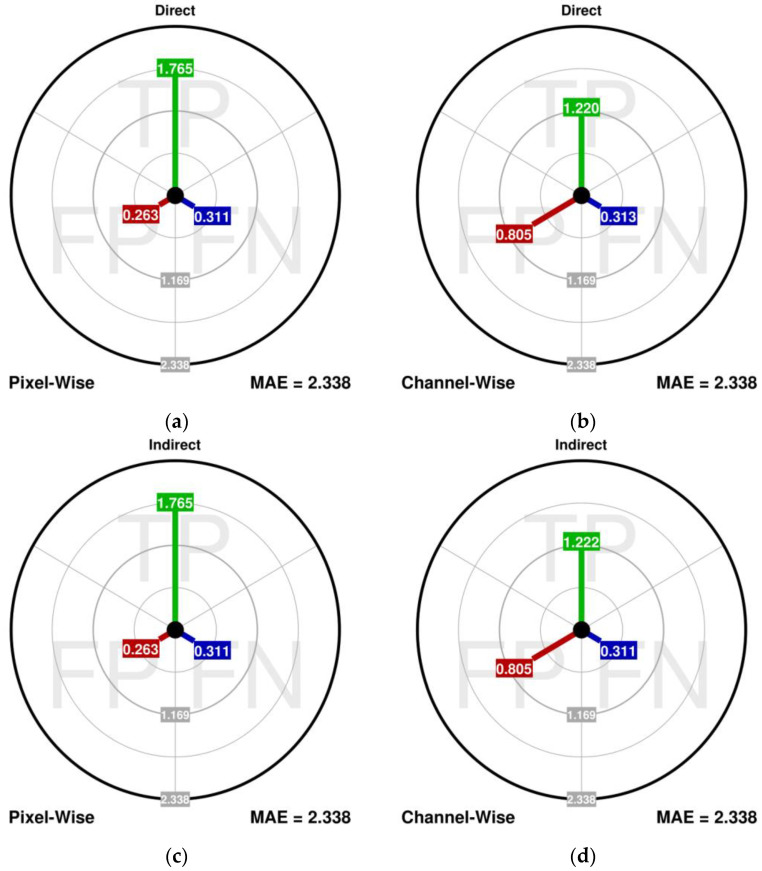
Difference between the PW (**a**,**c**) and CW analysis (**b**,**d**).

In the last experiment, the FAST-IPN algorithm, tuned to maximize the PSNR, is used to suppress the CIRI-type noise in image no. 2 (30% noise fraction). This time, the dMAE is computed using both the pixel-wise (PW) and the channel-wise (CW) analysis (Figure 8). Additionally, both direct and indirect versions of the aim plots are also compared. The results can be summarized as follows:

- In the PW analysis, the most impactful component is the MAE_TP_, while the MAE_FP_ component is far less significant (in which the MAE_FP_-to-MAE_TP_ ratio is 0.15). This may lead to the conclusion that the detection performance is fairly satisfactory, and the most promising improvement should be sought using a superior correction algorithm.
- However, if the CW analysis is performed, it can be found that the MAE_FP_ component is far more impactful than in the PW (with MAE_FP_-to-MAE_TP_ ratio at 0.66). It has been expected because the CIRI noise is processed in the PW manner, so there is a significant number of unnecessary estimations of the noise-free channels. Nevertheless, this result clearly shows that for the CIRI type of noise, there is still a lot of improvement to be pursued in the detection part of the filter, and additional, more precise CW detection might be worth pursuing.
- Also in the PW analysis, the MAE_FP_ is lower than the MAE_FN_, which might encourage trials of the retuning of the filter to reduce the MAE_FN_. However, the CW analysis shows that the MAE_FN_ is in fact the least impactful component, which means that its further reduction should not be a priority in this case.
- Differences in results using direct and indirect versions are negligible, so both versions are viable.

It is indispensable to mention that the PW processing, performed by most filters, is not very suitable for the CIRI type of noise, because it always corrects all the pixel channels regardless of which of them are corrupted. Therefore, the CW analysis is far more adequate when showing the performance of the filters in the case of the CIRI, or another type of impulsive noise is present. Moreover, it must be pointed out that the commonly used scalar measures are incapable of detecting and measuring the impact of this issue. Even if the scalar measure could be computed for every channel separately, it will not show the problem, due to the uniform distribution of the unnecessary corrections among the channels in the CIRI noise.

### 3.4. Statistical Properties of dMAE (Experiment IV)

**Figure 9:**
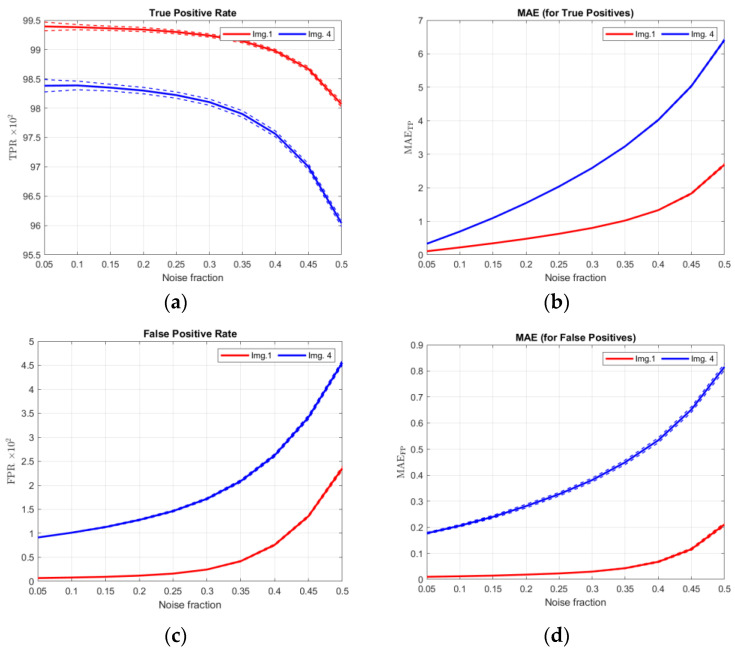

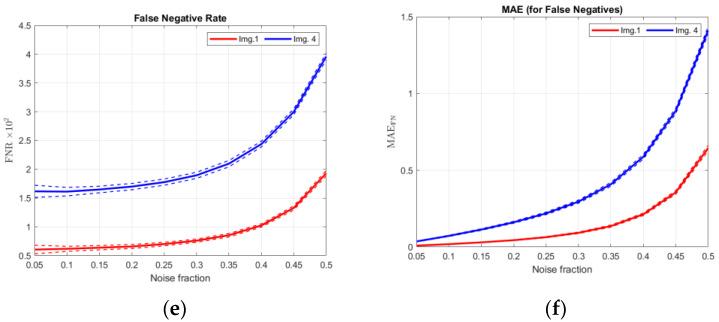
Comparison of TPR (**a**), FPR (**c**), and FNR (**e**) to components of dMAE (**b**,**d**,**f**) for images 1 and 4.

**Figure 10:**
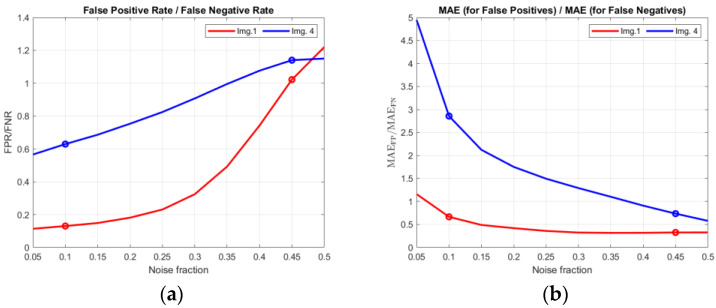
Comparison of FPR/FNR (**a**) ratio with MAE_FP_/MAE_FN_ (**b**) ratio (images 1 and 4).

Every image in the concerned image dataset is artificially corrupted 100 times using the impulsive noise CTRI of fractions taken from the set {0.05, 0.10, …, 0.50}. In the next step, all of them are filtered using the FAST-IPN algorithm, for the recommended value of its parameter ***T*** = 60. After that, the True Positive Rate—TPR (8), False Positive Rate—FPR (6), False Negative Rate—FNR (7), and dMAE (4) are computed for every detection/correction outcome. The results are grouped into sets by the image number, and for each set, means and standard deviations are obtained. It is important that the only variable factor in each set is a realization of noise; thus, properties of the image, algorithm, and *T* parameter do not influence the metric. Results (means ± standard deviation) for noise fractions 0.1, 0.2, …0.5, and all images, are presented in Table 5, Table 6, Table 7, Table 8, Table 9 and Table 10. Furthermore, results for images no. 1 and no. 4 are also presented in the plots in Figure 9. In Figure 10, ratios FPR/FNR and MAE_FP_/MAE_FN_ are also shown to present the advantage of using the dMAE.

(8) $$\mathrm{TPR}=\frac{\left|\mathrm{TP}\right|}{\left|\mathrm{TP}\right|+\left|\mathrm{FN}\right|}$$

If the values in Table 5, Table 6, Table 7, Table 8, Table 9 and Table 10 are taken into consideration, it can be stated that for both types of measures TPR/FPR/FNR and components of dMAE, the standard deviation is relatively small in every case. This means that the realization of the random noise does not influence the stability of these metrics. The main factor that strongly influences the values of dMAE components is the image itself. The filtration of more homogenous images (nos. 1, and 5) results in considerably lower values of dMAE components, while the values obtained for more heterogenic images (nos. 2 and 4) are significantly higher. This is a common behavior of many commonly used metrics such as PSNR, MSE, MAE, and NCD.

One of the main advantages of dMAE can be seen in Figure 9a,b. While TPR decreases only by approximately 1.0–2.0 percentage points from noise fraction 0.05 to 0.5 (depending on the image), MAE_TP_ increases even a few times for the same cases. This means that even if the cardinality of the errors does not change much, their impact on the image quality changes significantly. It is also presented in Figure 10 where ratios FPR/FNR and MAE_FP_/MAE_FN_ are shown. These can be commented on as follows:

- For image no. 1 and low noise fraction (0.1), there are about 9 times more FN pixels than FP pixels. However, the error introduced by FN pixels is only about 1.5 as high as that introduced by FP pixels—this can be deduced from the MAE_FP_/MAE_FN_ ratio.
- For the same image and high noise fraction (0.45), the number of FP pixels and the number of FN pixels are almost the same, and the error introduced by FN pixels still exceeds that introduced by FP pixels more than twice in magnitude.
- Both observations presented above show that for highly homogenous images, undetected noisy pixels (FN) are more damaging than those that are unnecessarily corrected (FP). It is a very intuitive behavior not reflected in classical FPR and FNR metrics but clearly seen using dMAE.
- For image no. 4 and low noise fraction (0.1), there is only about 1.5 more FN pixels than FP pixels, yet the error introduced by FP pixels is almost 3 times higher.
- For the same image and high noise fraction (0.45), the cardinality of FP pixels is about 10% higher than the cardinality of FN pixels, yet this time, FN pixels have about 1.3 more contributions in the error than FP pixels.
- For highly complex images, unnecessary corrections (FP) are far more damaging for low noise fractions, while for high ones, they damage the image almost the same as undetected noisy pixels (FN).

All those observations show that dMAE is a more informative matric than the TPR/FPR/FNR trio obtained using a simple confusion matrix because its components take into account not only cardinalities, but also actual damage caused by each class of pixels (TP/FP/FN) to the image.

## 4. Conclusions

The decomposed MAE is the hybrid of the image dissimilarity measure and detection performance measure. It enhances both by extending the first with more detailed information about possible sources of error, and by introducing actual processing context to the second. It is now possible to explicitly measure which kind of detection error (FP or FN) is more harmful to the output image quality. As decomposition is performed upon binary classification, this measure is suitable for the evaluation of color images (multi-channel) or gray-scale images (single-channel). Furthermore, for multi-channel images, both pixel-wise and channel-wise analyses are possible. The dMAE can be computed, even when maps of distortion (TM and DM) are not accessible.

The proposed form of presentation of decomposed MAE, the aim plots, are not only readable and intuitive but also allows for easy comparison of different results. Moreover, provided tools [44] are simple to use and provide an option to rapidly generate ready-to-publish aim plots. The alternative form of the aim plots capable of showing the results for multiple images on a single board remains an open problem for discussion and further development. The main issue here is that MAE for different images might significantly differ in value, and the normalization of the results reduces the decomposition effect of the measure, bringing it closer to the FRP and FNR measures.

It is difficult to dispute that dMAE is less useful as a direct performance index in the optimization of the algorithm’s parameters than scalar metrics, as it contains three components. However, it is a good tool for evaluating the outcome of such an optimization performed with another metric, as shown in the second experiment.

The proposed decomposed dissimilarity measure (based on MAE) and the form of presentation are not absolute and should be considered a starting point for this new type of performance analysis of denoising algorithms and other image correction problems, which do not process every pixel in the image. Moreover, as the application of scalar measures, such as the PSNR, MAE, and MSE, and the binary classification is not limited to image processing only, this type of decomposed measure and the aim plots might also be useful in very different tasks, such as removing artifacts from 3D scans and laser distance measurements.

## Figures and Tables

**Table 1:** Comparison of PSNR values.

No.	VMF	FPG-AMF	FAST-AMF	FAST-IPN
**1**	**32.33**	**34.43**	**35.53**	**39.17**
2	26.68	29.82	30.47	32.52
2	28.96	32.51	33.16	36.09
**4**	**24.16**	**27.69**	**28.44**	**30.13**
5	30.55	33.40	34.54	37.81

**Table 2:** Comparison of scalar MAE values.

No.	VMF	FPG-AMF	FAST-AMF	FAST-IPN
**1**	**2.45**	**0.84**	**0.72**	**0.54**
2	6.16	1.99	1.83	1.41
3	3.90	1.14	1.04	0.83
**4**	**8.38**	**2.77**	**2.55**	**1.99**
5	3.20	1.08	0.93	0.63

**Table 3:** Comparison of decomposed MAE values (MAE_TP_/MAE_FP_/MAE_FN_).

No.	VMF	FPG-AMF	FAST-AMF	FAST-IPN
**1**	**0.69/ 1.75/ 0.01**	**0.60/ 0.02/ 0.16**	**0.59/ 0.02/ 0.04**	**0.47/ 0.02/ 0.04**
2	1.75/ 4.41/ 0.02	1.47/ 0.24/ 0.23	1.47/ 0.19/ 0.11	1.14/ 0.16/ 0.11
3	1.13/ 2.73/ 0.03	0.84/ 0.05/ 0.16	0.83/ 0.06/ 0.06	0.71/ 0.05/ 0.06
**4**	**2.43/ 5.96/ 0.02**	**2.04/ 0.42/ 0.28**	**2.04/ 0.32/ 0.16**	**1.56/ 0.28/ 0.16**
5	0.99/ 2.22/ 0.01	0.78/ 0.04/ 0.20	0.78/ 0.03/ 0.06	0.54/ 0.03/ 0.06

**Table 4:** FAST-IPN Parameter tuning results.

	Tuned to Maximum MCC		Tuned to Maximum PNSR	
Noise Fraction	20%	40%	20%	40%
MCC (▲)	0.9931	0.9831	0.9920	0.9755
FPR (▼)	0.0016	0.0091	0.0026	0.0190
FNR (▼)	0.0048	0.0067	0.0022	0.0013
PSNR (▲)	39.4747	32.3078	39.7105	33.8293
MAE (▼)	0.5294	1.4931	0.5266	1.3942
NCD (▼)	40.6947	119.0729	39.3669	100.4373
FSIMc (▲)	0.9951	0.9689	0.9955	0.9777
dMAE:				
MAE_TP_ (▼)	0.4724	1.2693	0.4716	1.2025
MAE_FP_ (▼)	0.0248	0.0768	0.0397	0.1591
MAE_FN_ (▼)	0.0316	0.1442	0.0152	0.0326
Parameter T value (def. 60)	**55**	**52**	**47**	**33**

▲ Higher value is better. ▼ Lower value is better.

**Table 5:** Statistics for *TPR* (mean + std) for different noise fractions.

No.	TPR (×10^2^)				
	0.1	0.2	0.3	0.4	0.5
**1**	**99.382 ± 0.044**	**99.342 ± 0.035**	**99.240 ± 0.028**	**98.976 ± 0.029**	**98.070 ± 0.046**
2	98.711 ± 0.066	98.642 ± 0.044	98.475 ± 0.042	98.031 ± 0.047	96.738 ± 0.050
3	99.229 ± 0.047	99.173 ± 0.034	99.043 ± 0.033	98.705 ± 0.037	97.581 ± 0.044
**4**	**98.389 ± 0.074**	**98.301 ± 0.055**	**98.104 ± 0.053**	**97.562 ± 0.050**	**96.041 ± 0.059**
5	99.139 ± 0.054	99.102 ± 0.037	98.998 ± 0.041	98.696 ± 0.036	97.705 ± 0.045

**Table 6:** Statistics for MAE_TP_ (mean + std) for different noise fractions.

No.	MAE_TP_				
	0.1	0.2	0.3	0.4	0.5
**1**	**0.220 ± 0.002**	**0.477 ± 0.004**	**0.801 ± 0.007**	**1.333 ± 0.015**	**2.692 ± 0.037**
2	0.517 ± 0.004	1.132 ± 0.007	1.885 ± 0.011	2.943 ± 0.019	4.890 ± 0.036
3	0.325 ± 0.003	0.703 ± 0.005	1.169 ± 0.009	1.922 ± 0.022	3.832 ± 0.044
**4**	**0.696 ± 0.006**	**1.545 ± 0.011**	**2.592 ± 0.013**	**4.024 ± 0.023**	**6.410 ± 0.036**
5	0.245 ± 0.003	0.536 ± 0.005	0.904 ± 0.008	1.491 ± 0.014	2.870 ± 0.030

**Table 7:** Statistics for *FPR* (mean + std) for different noise fractions.

No.	FPR (×10^2^)				
	0.1	0.2	0.3	0.4	0.5
**1**	**0.081 ± 0.004**	**0.119 ± 0.006**	**0.246 ± 0.010**	**0.760 ± 0.019**	**2.355 ± 0.042**
2	0.508 ± 0.009	0.666 ± 0.013	0.951 ± 0.016	1.647 ± 0.030	3.339 ± 0.044
2	0.175 ± 0.005	0.231 ± 0.007	0.384 ± 0.011	0.974 ± 0.022	2.807 ± 0.043
**4**	**1.014 ± 0.010**	**1.280 ± 0.017**	**1.720 ± 0.022**	**2.624 ± 0.033**	**4.554 ± 0.048**
5	0.119 ± 0.005	0.186 ± 0.008	0.352 ± 0.013	0.888 ± 0.021	2.400 ± 0.041

**Table 8:** Statistics for MAE_FP_ (mean + std) for different noise fractions.

No.	MAE_FP_				
	0.1	0.2	0.3	0.4	0.5
**1**	**0.012 ± 0.001**	**0.018 ± 0.002**	**0.030 ± 0.002**	**0.068 ± 0.003**	**0.211 ± 0.006**
2	0.128 ± 0.004	0.160 ± 0.005	0.206 ± 0.005	0.293 ± 0.007	0.498 ± 0.010
3	0.038 ± 0.002	0.048 ± 0.002	0.065 ± 0.003	0.121 ± 0.005	0.350 ± 0.009
**4**	**0.206 ± 0.005**	**0.281 ± 0.007**	**0.381 ± 0.008**	**0.534 ± 0.010**	**0.815 ± 0.012**
5	0.017 ± 0.001	0.027 ± 0.002	0.045 ± 0.002	0.092 ± 0.004	0.230 ± 0.006

**Table 9:** Statistics for *FNR* (mean + std) for different noise fractions.

No.	FNR (×10^2^)				
	0.1	0.2	0.3	0.4	0.5
**1**	**0.618 ± 0.044**	**0.658 ± 0.035**	**0.760 ± 0.028**	**1.024 ± 0.029**	**1.930 ± 0.046**
2	1.289 ± 0.066	1.358 ± 0.044	1.525 ± 0.042	1.969 ± 0.047	3.262 ± 0.050
3	0.771 ± 0.047	0.827 ± 0.034	0.957 ± 0.033	1.295 ± 0.037	2.419 ± 0.044
**4**	**1.611 ± 0.074**	**1.699 ± 0.055**	**1.896 ± 0.053**	**2.438 ± 0.050**	**3.959 ± 0.059**
5	0.861 ± 0.054	0.898 ± 0.037	1.002 ± 0.041	1.304 ± 0.036	2.295 ± 0.045

**Table 10:** Statistics for MAE_FN_ (mean + std) for different noise fractions.

No.	MAE_FN_				
	0.1	0.2	0.3	0.4	0.5
**1**	**0.018 ± 0.002**	**0.044 ± 0.003**	**0.092 ± 0.005**	**0.212 ± 0.008**	**0.644 ± 0.019**
2	0.048 ± 0.003	0.110 ± 0.005	0.209 ± 0.007	0.432 ± 0.013	1.090 ± 0.022
3	0.026 ± 0.002	0.063 ± 0.003	0.133 ± 0.006	0.310 ± 0.011	0.948 ± 0.021
**4**	**0.072 ± 0.004**	**0.161 ± 0.006**	**0.295 ± 0.010**	**0.587 ± 0.015**	**1.410 ± 0.024**
5	0.028 ± 0.002	0.064 ± 0.003	0.122 ± 0.006	0.258 ± 0.009	0.711 ± 0.016

## Data Availability

Matlab functions for computing dMAE and creating aim plots, image data sets (original, corrupted, and restored images) for all experiments (Section 3.1, Section 3.2 and Section 3.3), and noise maps for the 3rd experiment (Section 3.3) are available from Supplementary Materials and here: https://ch.mathworks.com/matlabcentral/fileexchange/112290-aim-plots (accessed on 1 April 2023).

## Supplementary Materials

The following supporting information can be downloaded at: https://www.mdpi.com/article/10.3390/s23125657/s1.
