# Supplementary figures and images for: Decomposed Dissimilarity Measure for Evaluation of Digital Image Denoising
Source: Sensors (Basel). 2023 Jun 16;23(12):5657. doi: 10.3390/s23125657 (PMC10304227; doi:10.3390/s23125657)

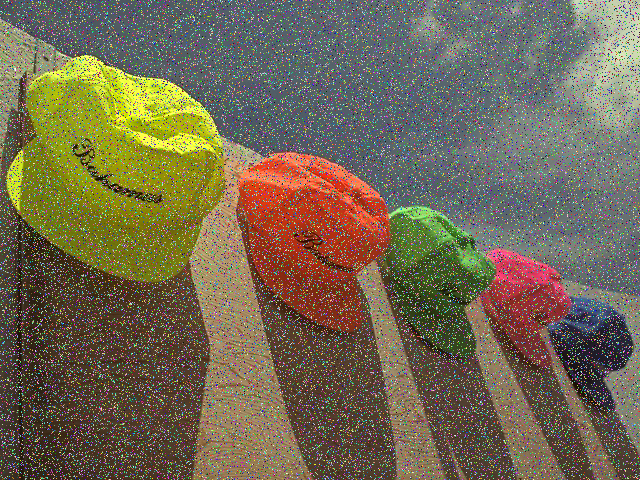

Supplement: Supplementary file 1 [file sensors-23-05657-s001.zip › sensors-2219351-supplementary/Sup_data/Exp_1_2/Corrupted/0.2/img01.png]

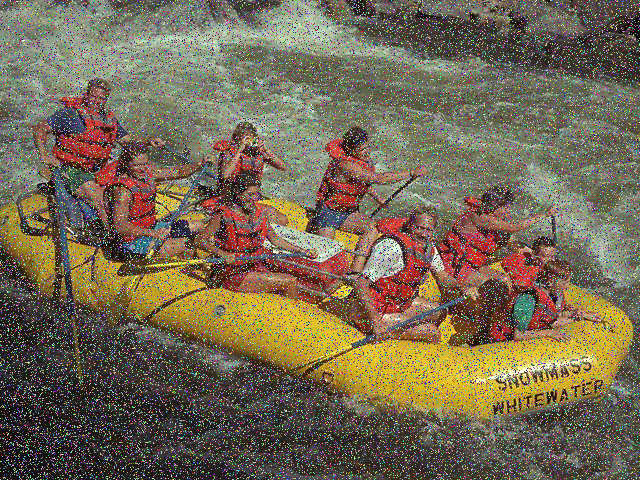

Supplement: Supplementary file 1 [file sensors-23-05657-s001.zip › sensors-2219351-supplementary/Sup_data/Exp_1_2/Corrupted/0.2/img02.png]

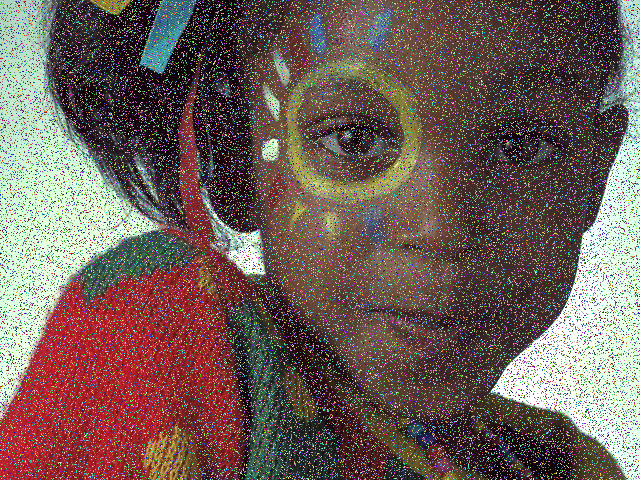

Supplement: Supplementary file 1 [file sensors-23-05657-s001.zip › sensors-2219351-supplementary/Sup_data/Exp_1_2/Corrupted/0.2/img03.png]

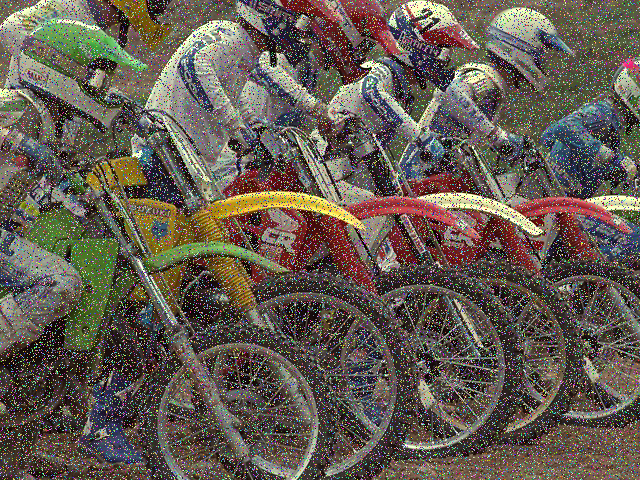

Supplement: Supplementary file 1 [file sensors-23-05657-s001.zip › sensors-2219351-supplementary/Sup_data/Exp_1_2/Corrupted/0.2/img04.png]

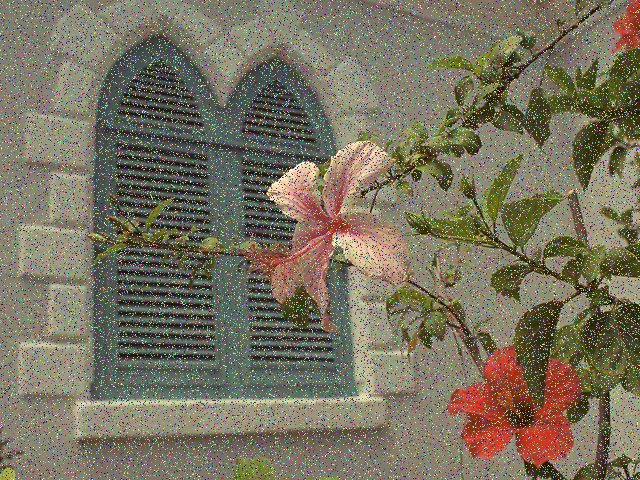

Supplement: Supplementary file 1 [file sensors-23-05657-s001.zip › sensors-2219351-supplementary/Sup_data/Exp_1_2/Corrupted/0.2/img05.png]

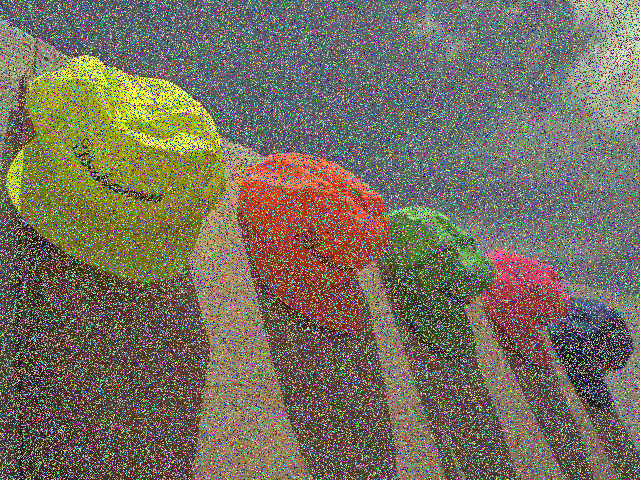

Supplement: Supplementary file 1 [file sensors-23-05657-s001.zip › sensors-2219351-supplementary/Sup_data/Exp_1_2/Corrupted/0.4/img01.png]

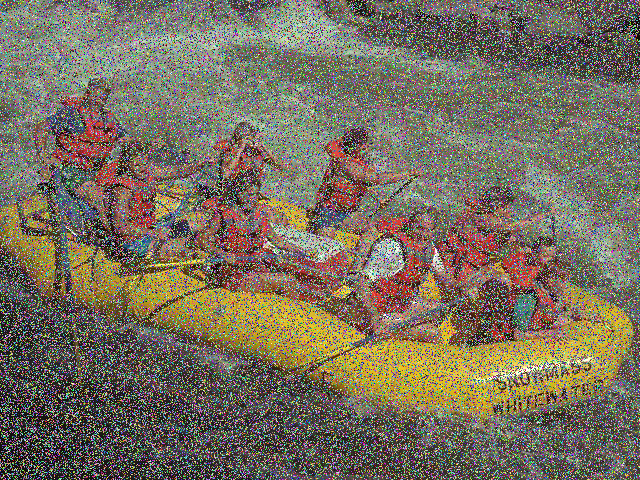

Supplement: Supplementary file 1 [file sensors-23-05657-s001.zip › sensors-2219351-supplementary/Sup_data/Exp_1_2/Corrupted/0.4/img02.png]

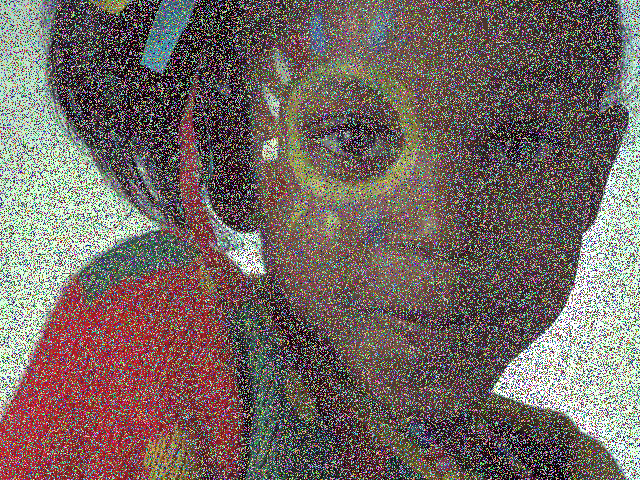

Supplement: Supplementary file 1 [file sensors-23-05657-s001.zip › sensors-2219351-supplementary/Sup_data/Exp_1_2/Corrupted/0.4/img03.png]

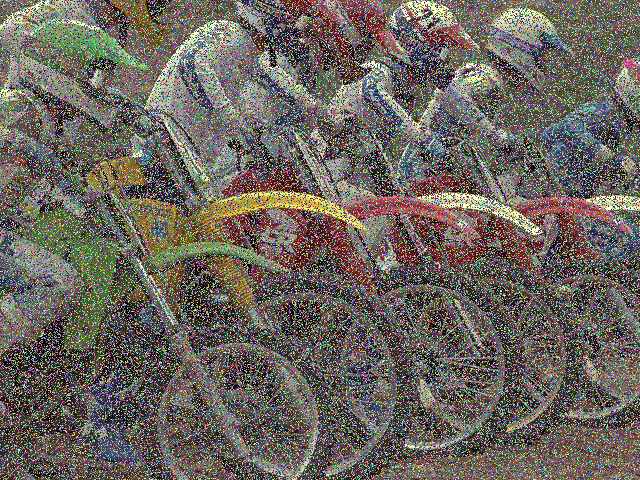

Supplement: Supplementary file 1 [file sensors-23-05657-s001.zip › sensors-2219351-supplementary/Sup_data/Exp_1_2/Corrupted/0.4/img04.png]

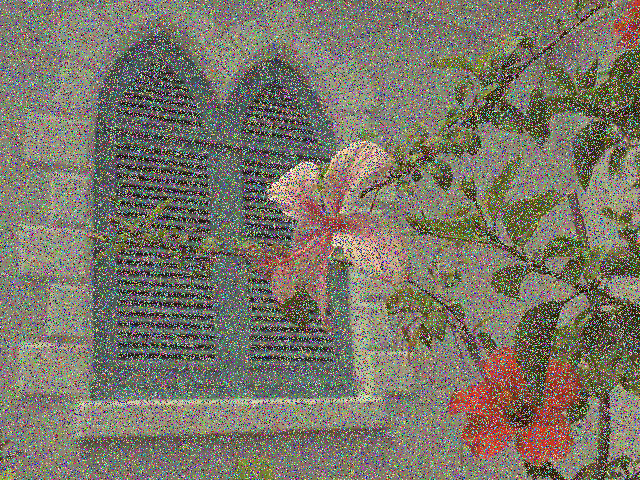

Supplement: Supplementary file 1 [file sensors-23-05657-s001.zip › sensors-2219351-supplementary/Sup_data/Exp_1_2/Corrupted/0.4/img05.png]

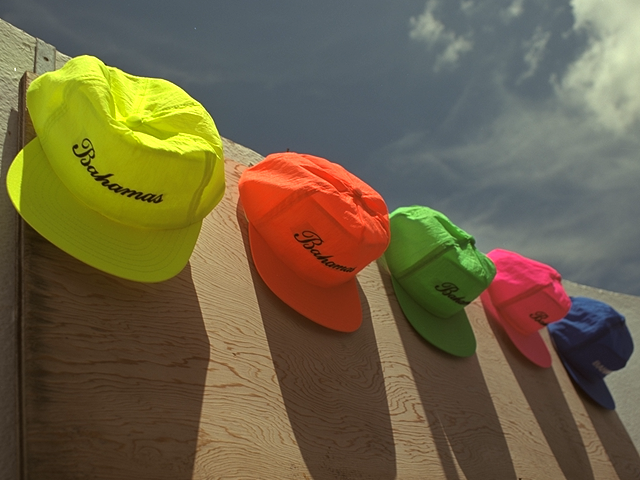

Supplement: Supplementary file 1 [file sensors-23-05657-s001.zip › sensors-2219351-supplementary/Sup_data/Exp_1_2/Orginal/pic01.png]

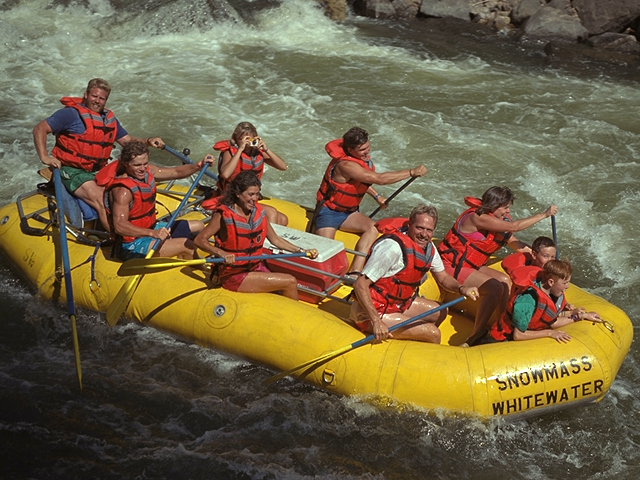

Supplement: Supplementary file 1 [file sensors-23-05657-s001.zip › sensors-2219351-supplementary/Sup_data/Exp_1_2/Orginal/pic02.png]

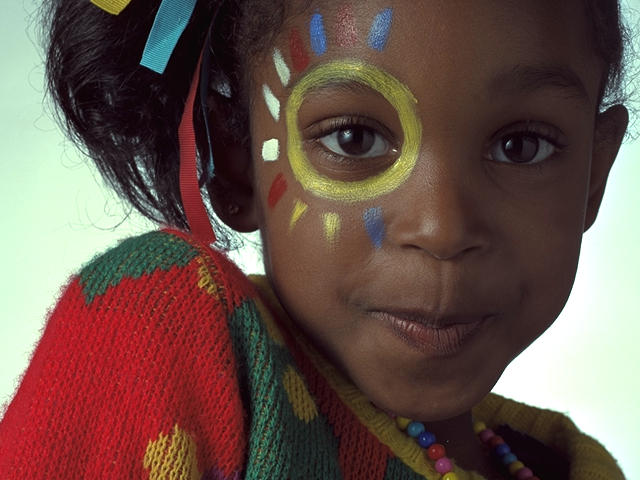

Supplement: Supplementary file 1 [file sensors-23-05657-s001.zip › sensors-2219351-supplementary/Sup_data/Exp_1_2/Orginal/pic03.png]

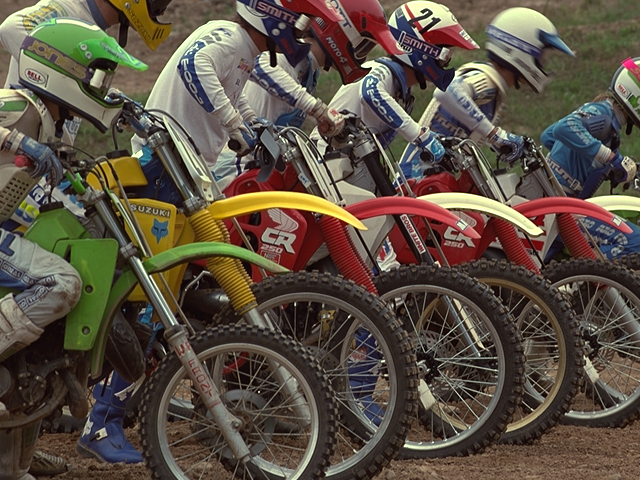

Supplement: Supplementary file 1 [file sensors-23-05657-s001.zip › sensors-2219351-supplementary/Sup_data/Exp_1_2/Orginal/pic04.png]

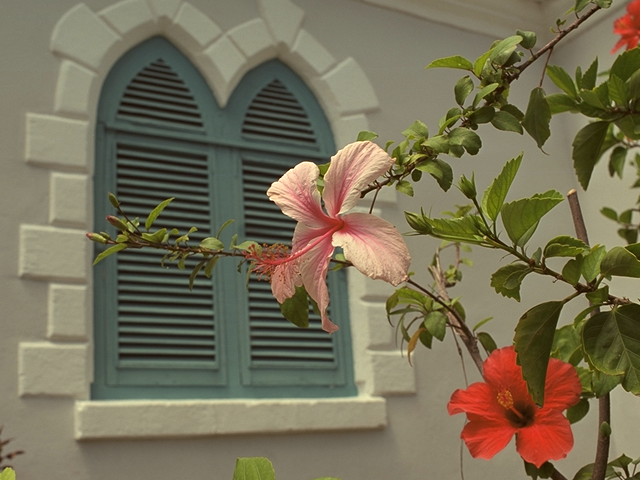

Supplement: Supplementary file 1 [file sensors-23-05657-s001.zip › sensors-2219351-supplementary/Sup_data/Exp_1_2/Orginal/pic05.png]

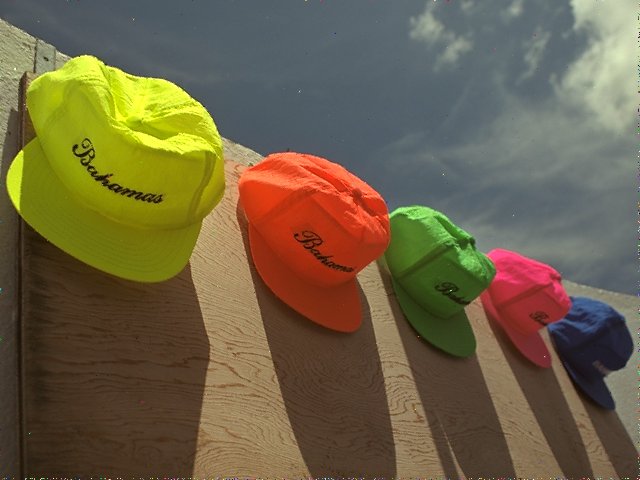

Supplement: Supplementary file 1 [file sensors-23-05657-s001.zip › sensors-2219351-supplementary/Sup_data/Exp_1_2/Restored/0.2/FAST-AMF/img01.png]

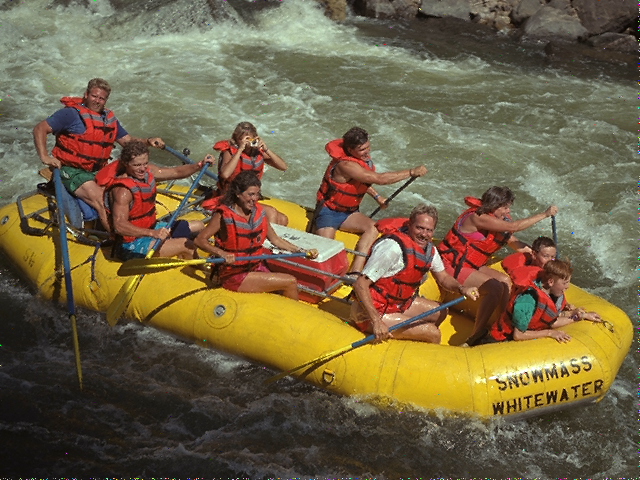

Supplement: Supplementary file 1 [file sensors-23-05657-s001.zip › sensors-2219351-supplementary/Sup_data/Exp_1_2/Restored/0.2/FAST-AMF/img02.png]

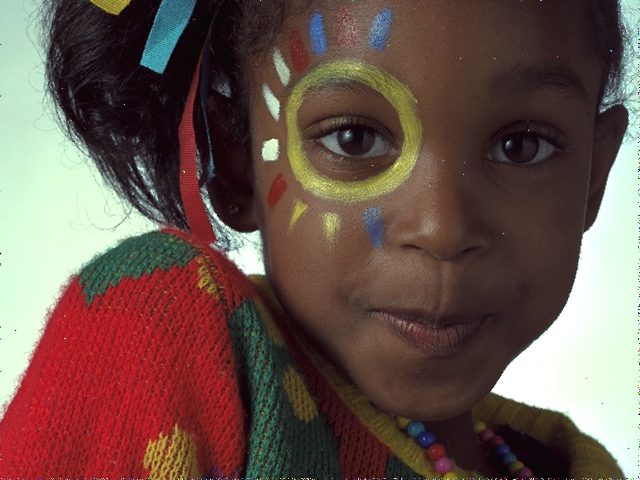

Supplement: Supplementary file 1 [file sensors-23-05657-s001.zip › sensors-2219351-supplementary/Sup_data/Exp_1_2/Restored/0.2/FAST-AMF/img03.png]

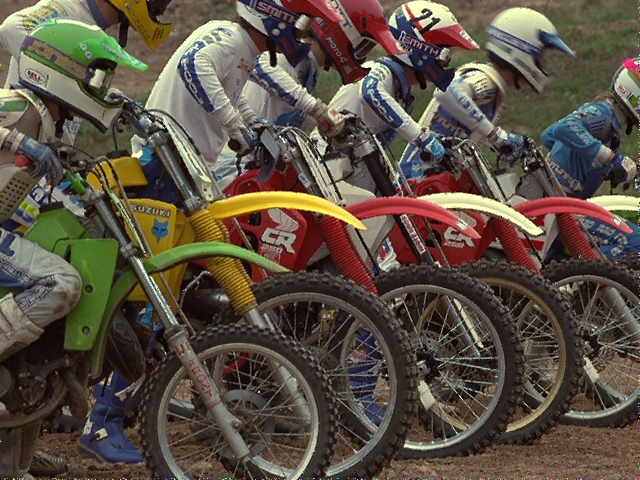

Supplement: Supplementary file 1 [file sensors-23-05657-s001.zip › sensors-2219351-supplementary/Sup_data/Exp_1_2/Restored/0.2/FAST-AMF/img04.png]

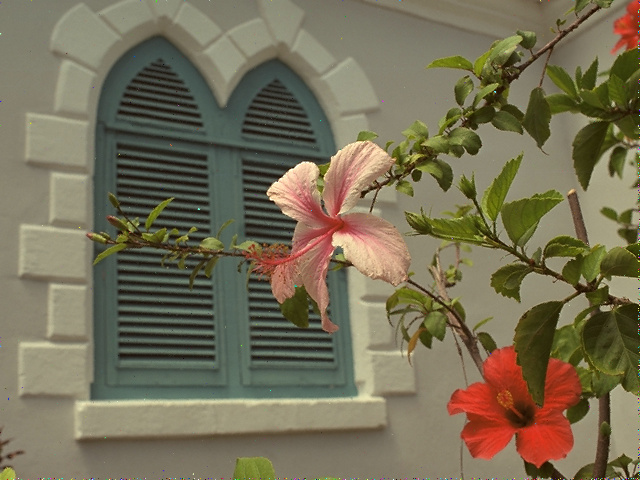

Supplement: Supplementary file 1 [file sensors-23-05657-s001.zip › sensors-2219351-supplementary/Sup_data/Exp_1_2/Restored/0.2/FAST-AMF/img05.png]

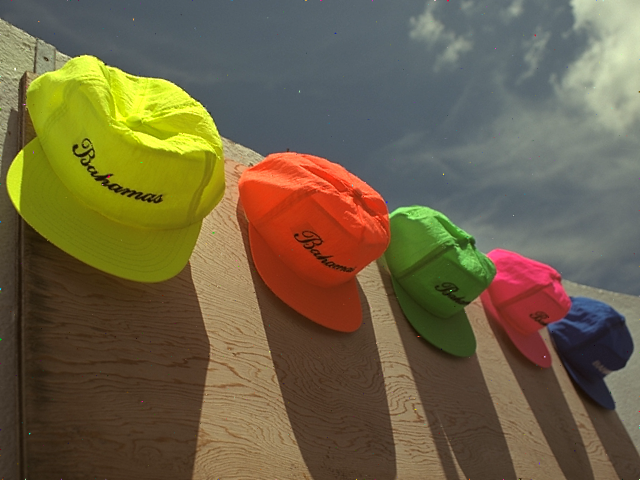

Supplement: Supplementary file 1 [file sensors-23-05657-s001.zip › sensors-2219351-supplementary/Sup_data/Exp_1_2/Restored/0.2/FAST-IPN/img01.png]

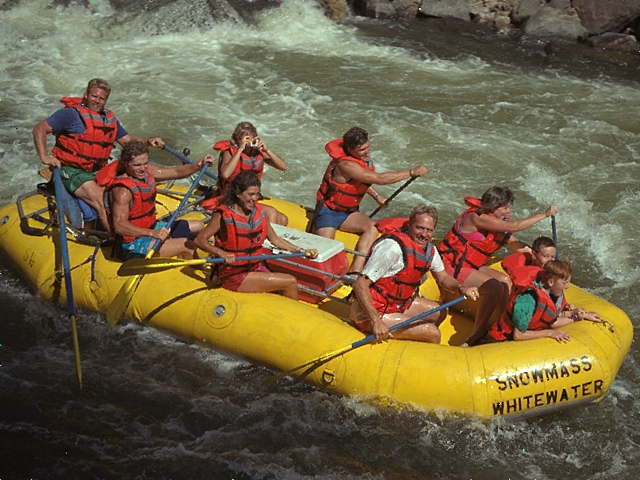

Supplement: Supplementary file 1 [file sensors-23-05657-s001.zip › sensors-2219351-supplementary/Sup_data/Exp_1_2/Restored/0.2/FAST-IPN/img02.png]

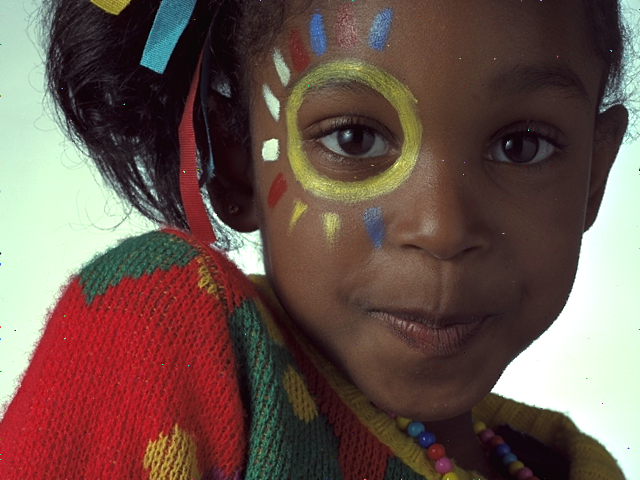

Supplement: Supplementary file 1 [file sensors-23-05657-s001.zip › sensors-2219351-supplementary/Sup_data/Exp_1_2/Restored/0.2/FAST-IPN/img03.png]

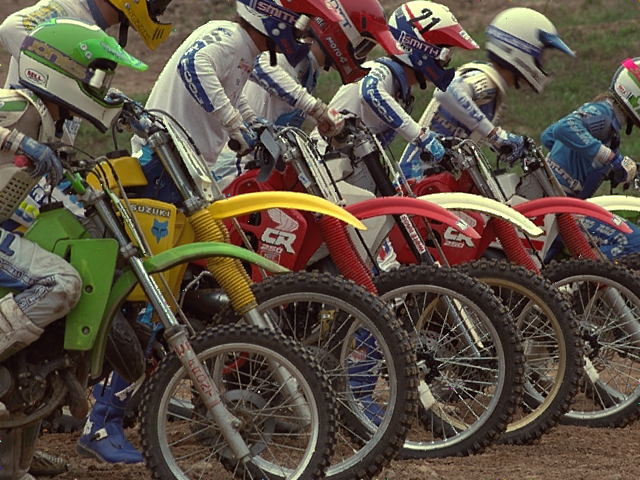

Supplement: Supplementary file 1 [file sensors-23-05657-s001.zip › sensors-2219351-supplementary/Sup_data/Exp_1_2/Restored/0.2/FAST-IPN/img04.png]

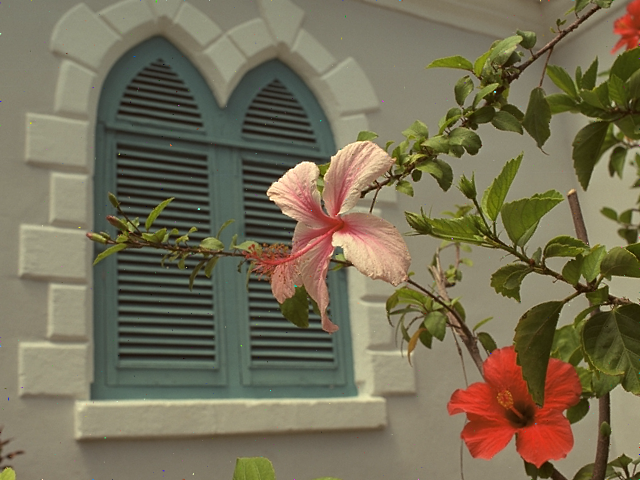

Supplement: Supplementary file 1 [file sensors-23-05657-s001.zip › sensors-2219351-supplementary/Sup_data/Exp_1_2/Restored/0.2/FAST-IPN/img05.png]

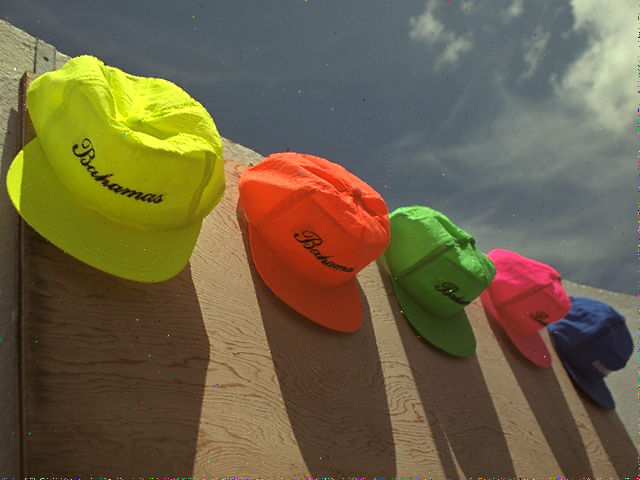

Supplement: Supplementary file 1 [file sensors-23-05657-s001.zip › sensors-2219351-supplementary/Sup_data/Exp_1_2/Restored/0.2/FPG-AMF/img01.png]

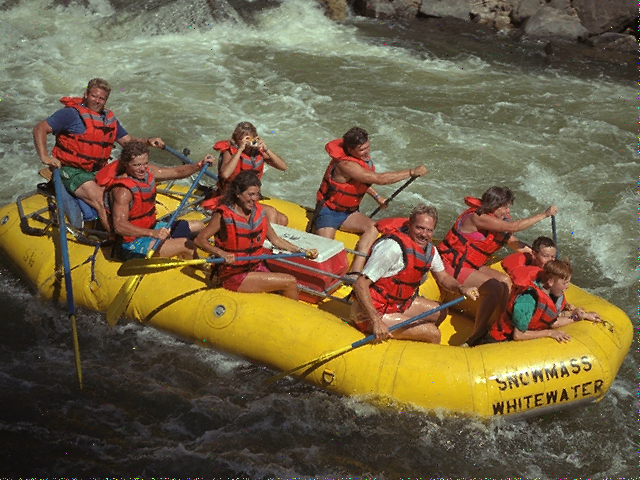

Supplement: Supplementary file 1 [file sensors-23-05657-s001.zip › sensors-2219351-supplementary/Sup_data/Exp_1_2/Restored/0.2/FPG-AMF/img02.png]

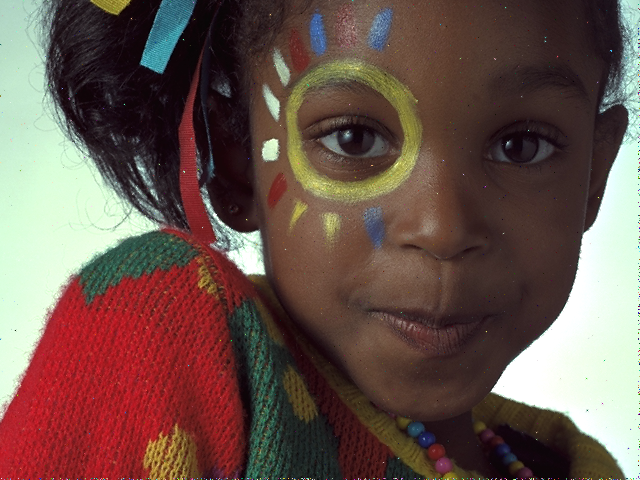

Supplement: Supplementary file 1 [file sensors-23-05657-s001.zip › sensors-2219351-supplementary/Sup_data/Exp_1_2/Restored/0.2/FPG-AMF/img03.png]

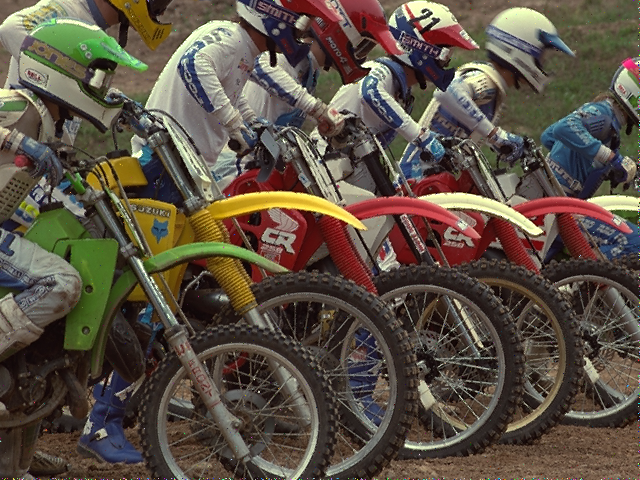

Supplement: Supplementary file 1 [file sensors-23-05657-s001.zip › sensors-2219351-supplementary/Sup_data/Exp_1_2/Restored/0.2/FPG-AMF/img04.png]

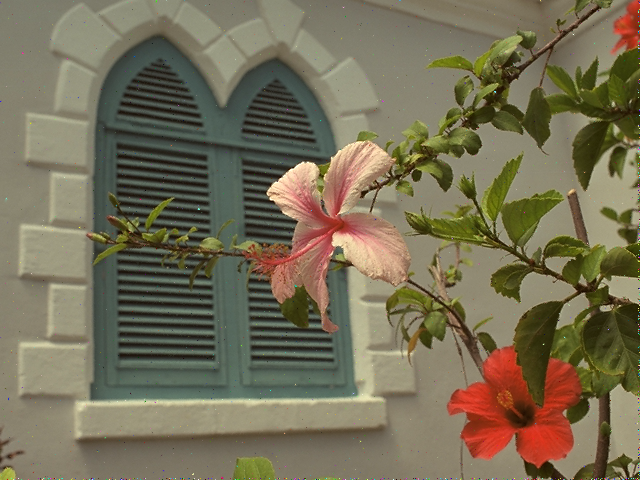

Supplement: Supplementary file 1 [file sensors-23-05657-s001.zip › sensors-2219351-supplementary/Sup_data/Exp_1_2/Restored/0.2/FPG-AMF/img05.png]

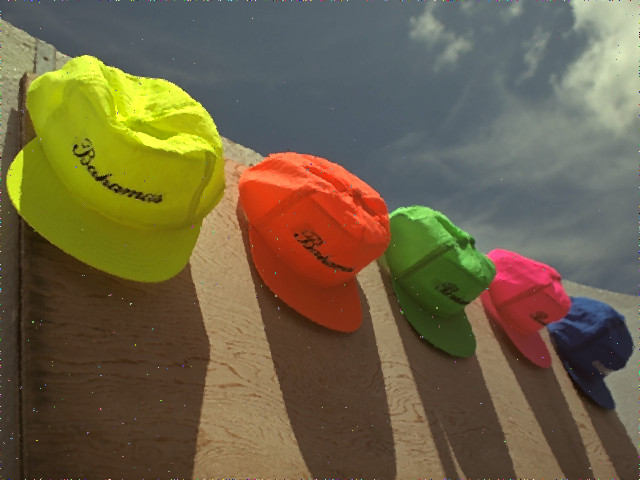

Supplement: Supplementary file 1 [file sensors-23-05657-s001.zip › sensors-2219351-supplementary/Sup_data/Exp_1_2/Restored/0.2/VMF/img01.png]

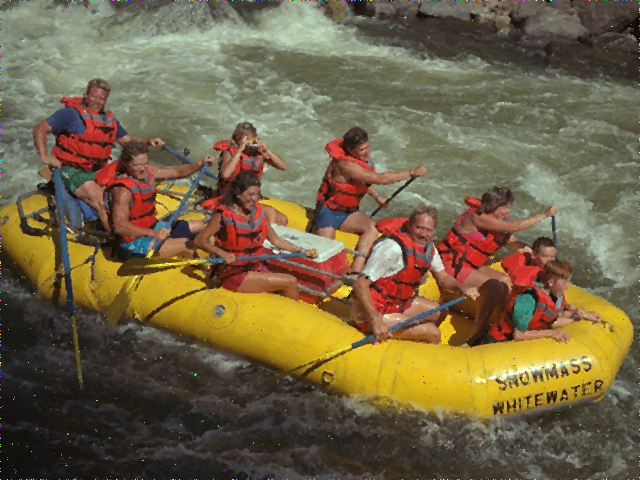

Supplement: Supplementary file 1 [file sensors-23-05657-s001.zip › sensors-2219351-supplementary/Sup_data/Exp_1_2/Restored/0.2/VMF/img02.png]

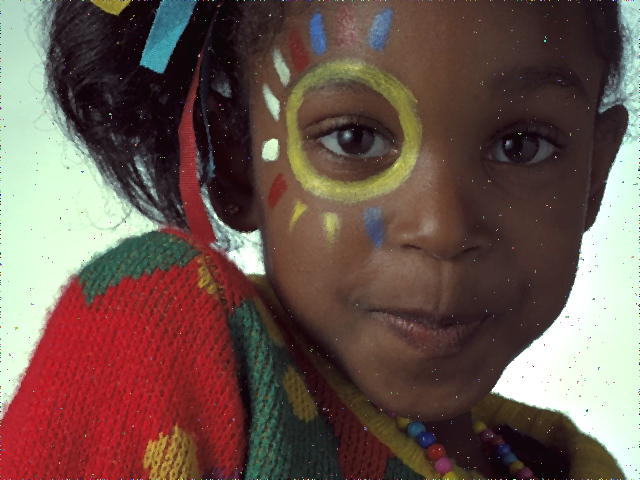

Supplement: Supplementary file 1 [file sensors-23-05657-s001.zip › sensors-2219351-supplementary/Sup_data/Exp_1_2/Restored/0.2/VMF/img03.png]

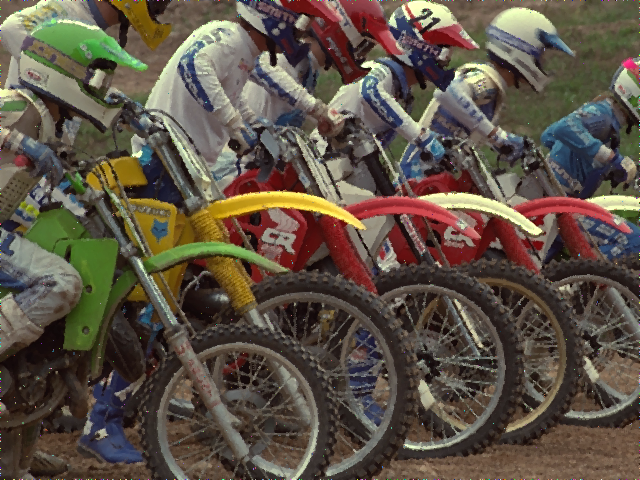

Supplement: Supplementary file 1 [file sensors-23-05657-s001.zip › sensors-2219351-supplementary/Sup_data/Exp_1_2/Restored/0.2/VMF/img04.png]

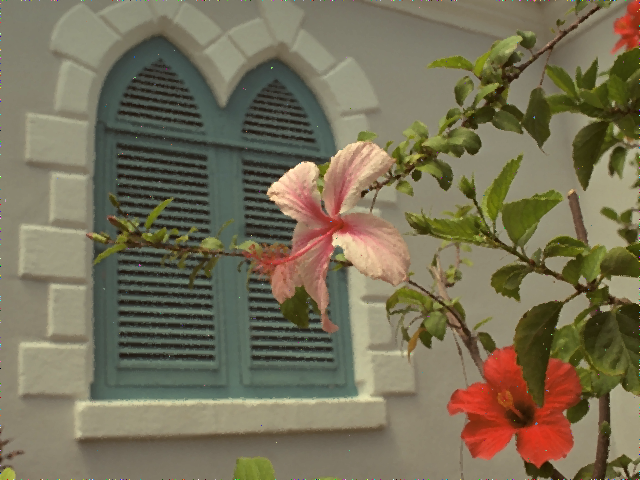

Supplement: Supplementary file 1 [file sensors-23-05657-s001.zip › sensors-2219351-supplementary/Sup_data/Exp_1_2/Restored/0.2/VMF/img05.png]

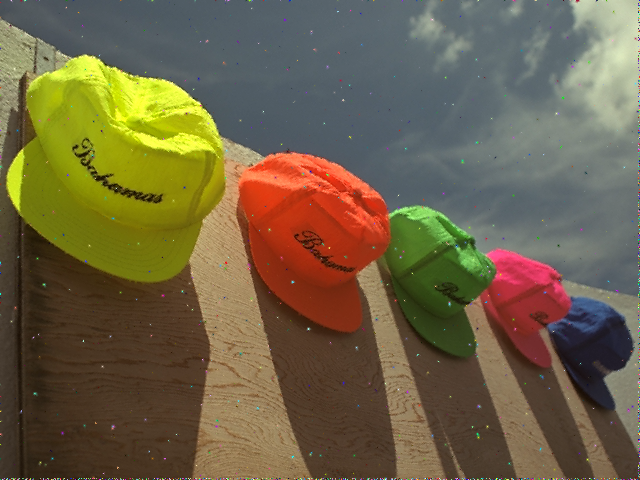

Supplement: Supplementary file 1 [file sensors-23-05657-s001.zip › sensors-2219351-supplementary/Sup_data/Exp_1_2/Restored/0.4/FAST-AMF/img01.png]

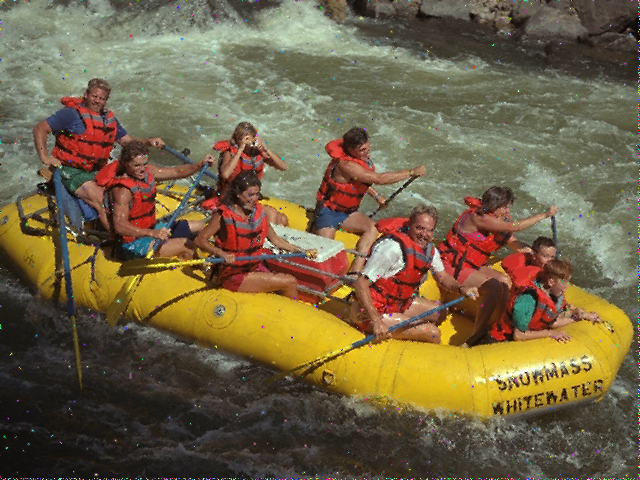

Supplement: Supplementary file 1 [file sensors-23-05657-s001.zip › sensors-2219351-supplementary/Sup_data/Exp_1_2/Restored/0.4/FAST-AMF/img02.png]

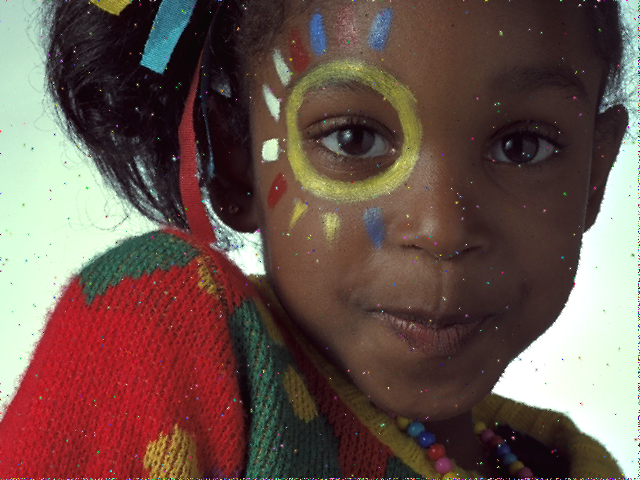

Supplement: Supplementary file 1 [file sensors-23-05657-s001.zip › sensors-2219351-supplementary/Sup_data/Exp_1_2/Restored/0.4/FAST-AMF/img03.png]

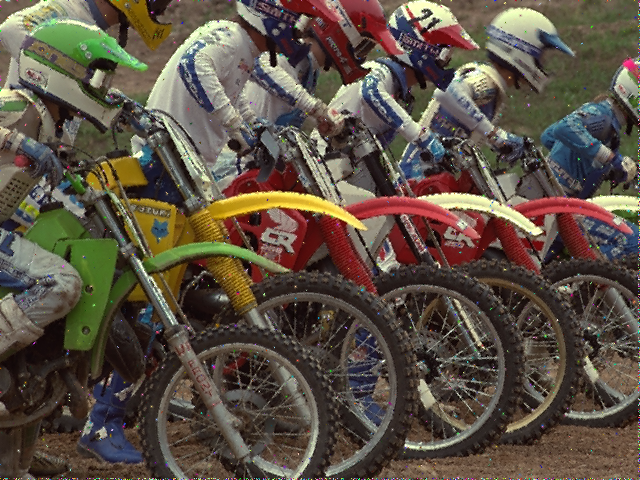

Supplement: Supplementary file 1 [file sensors-23-05657-s001.zip › sensors-2219351-supplementary/Sup_data/Exp_1_2/Restored/0.4/FAST-AMF/img04.png]

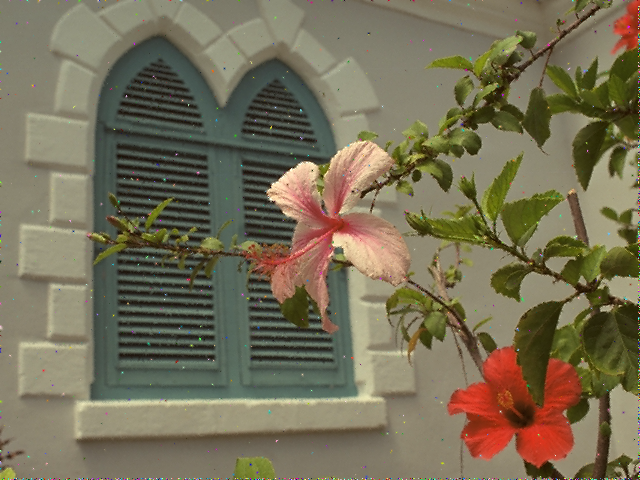

Supplement: Supplementary file 1 [file sensors-23-05657-s001.zip › sensors-2219351-supplementary/Sup_data/Exp_1_2/Restored/0.4/FAST-AMF/img05.png]

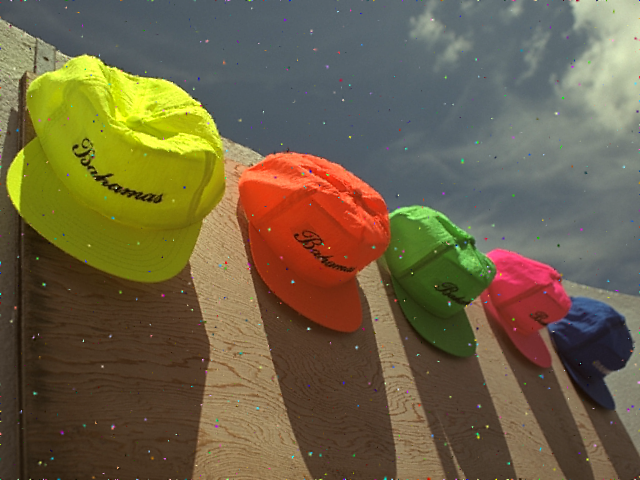

Supplement: Supplementary file 1 [file sensors-23-05657-s001.zip › sensors-2219351-supplementary/Sup_data/Exp_1_2/Restored/0.4/FAST-IPN/img01.png]

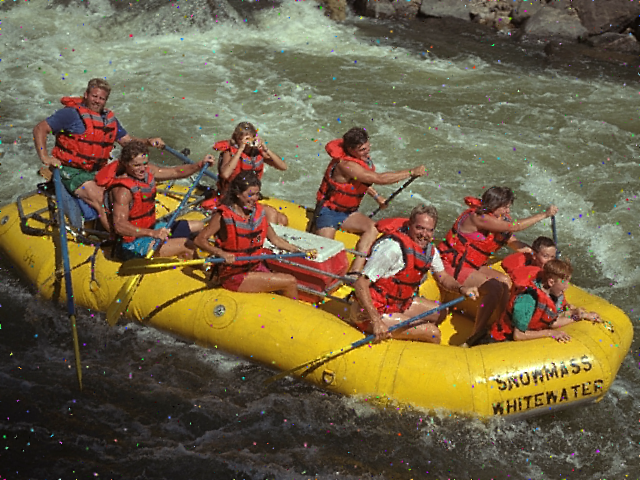

Supplement: Supplementary file 1 [file sensors-23-05657-s001.zip › sensors-2219351-supplementary/Sup_data/Exp_1_2/Restored/0.4/FAST-IPN/img02.png]

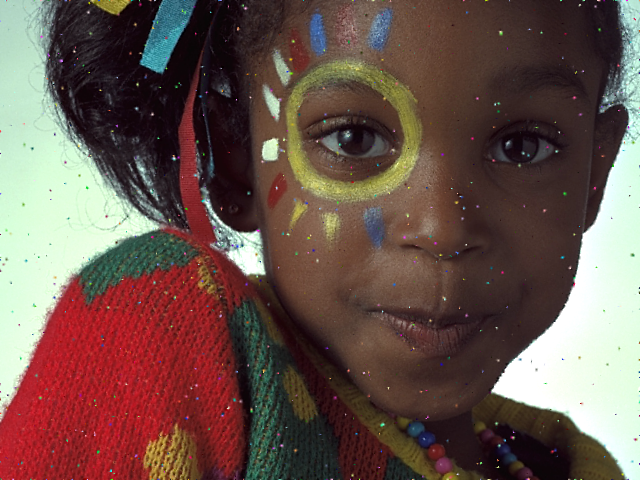

Supplement: Supplementary file 1 [file sensors-23-05657-s001.zip › sensors-2219351-supplementary/Sup_data/Exp_1_2/Restored/0.4/FAST-IPN/img03.png]

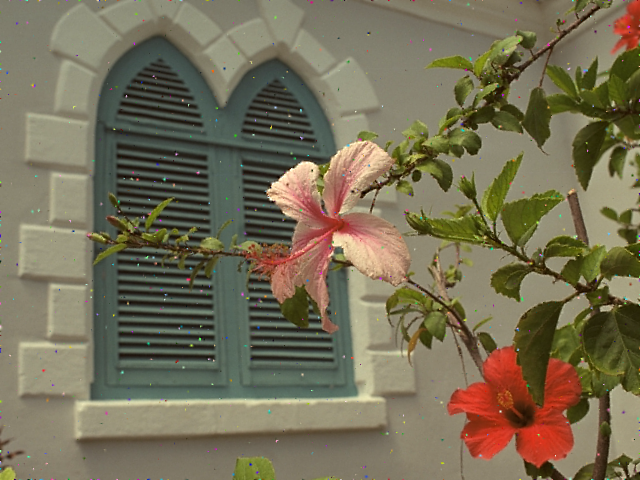

Supplement: Supplementary file 1 [file sensors-23-05657-s001.zip › sensors-2219351-supplementary/Sup_data/Exp_1_2/Restored/0.4/FAST-IPN/img04.png]

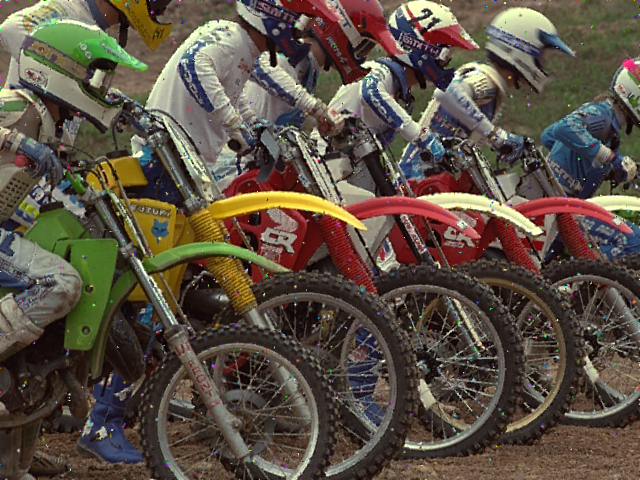

Supplement: Supplementary file 1 [file sensors-23-05657-s001.zip › sensors-2219351-supplementary/Sup_data/Exp_1_2/Restored/0.4/FAST-IPN/img05.png]

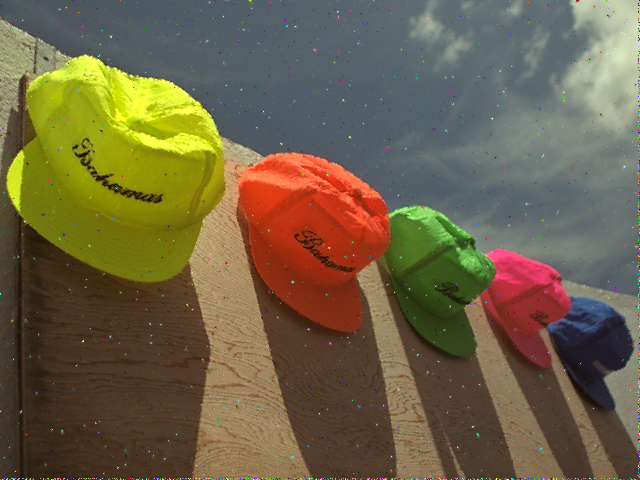

Supplement: Supplementary file 1 [file sensors-23-05657-s001.zip › sensors-2219351-supplementary/Sup_data/Exp_1_2/Restored/0.4/FPG-AMF/img01.png]

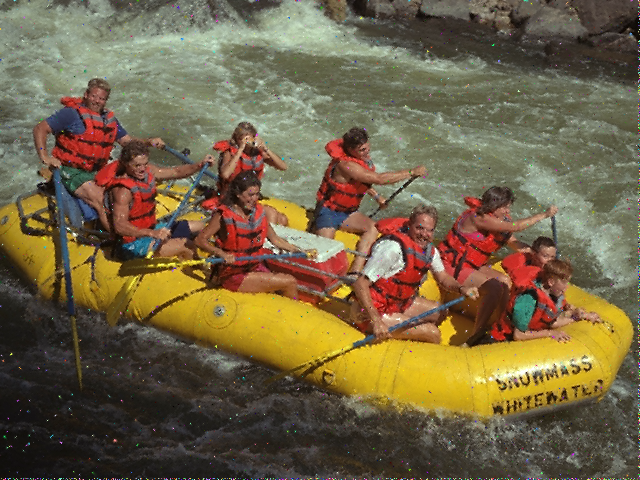

Supplement: Supplementary file 1 [file sensors-23-05657-s001.zip › sensors-2219351-supplementary/Sup_data/Exp_1_2/Restored/0.4/FPG-AMF/img02.png]

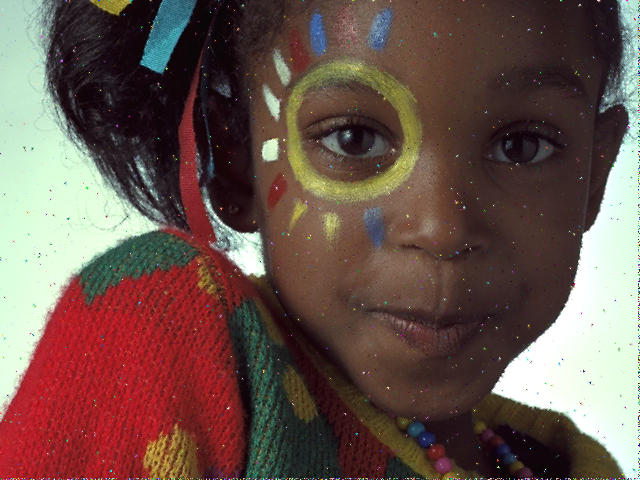

Supplement: Supplementary file 1 [file sensors-23-05657-s001.zip › sensors-2219351-supplementary/Sup_data/Exp_1_2/Restored/0.4/FPG-AMF/img03.png]

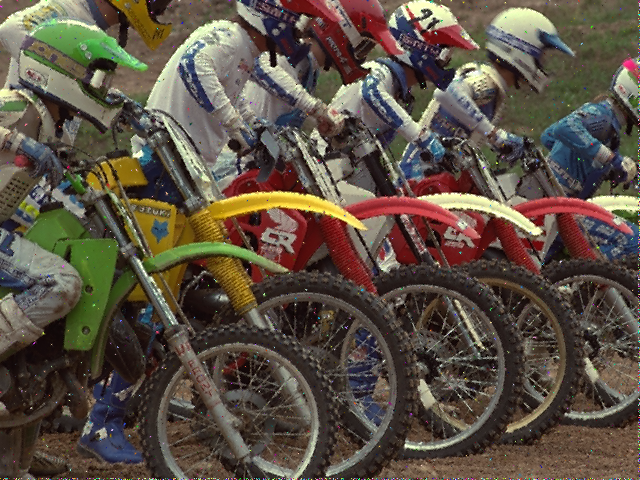

Supplement: Supplementary file 1 [file sensors-23-05657-s001.zip › sensors-2219351-supplementary/Sup_data/Exp_1_2/Restored/0.4/FPG-AMF/img04.png]

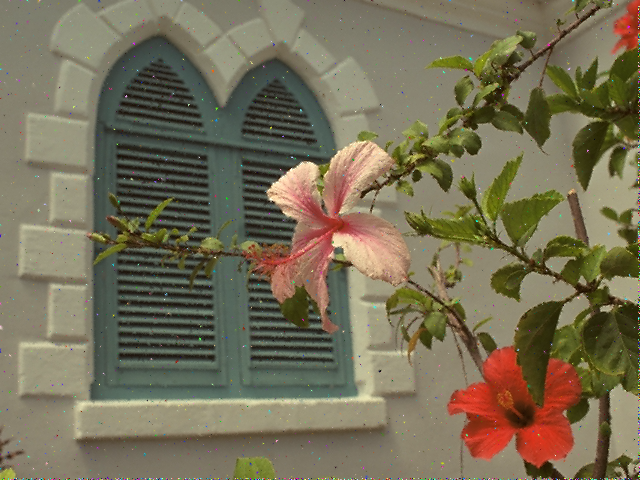

Supplement: Supplementary file 1 [file sensors-23-05657-s001.zip › sensors-2219351-supplementary/Sup_data/Exp_1_2/Restored/0.4/FPG-AMF/img05.png]

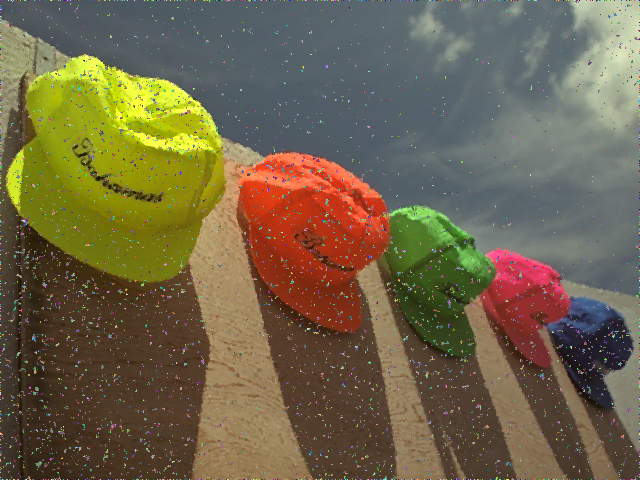

Supplement: Supplementary file 1 [file sensors-23-05657-s001.zip › sensors-2219351-supplementary/Sup_data/Exp_1_2/Restored/0.4/VMF/img01.png]

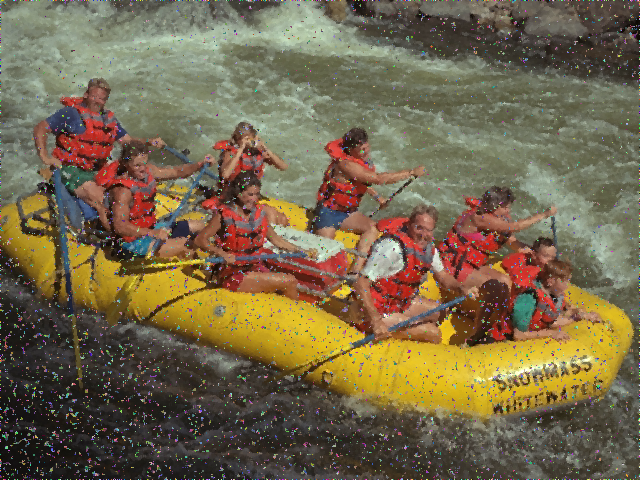

Supplement: Supplementary file 1 [file sensors-23-05657-s001.zip › sensors-2219351-supplementary/Sup_data/Exp_1_2/Restored/0.4/VMF/img02.png]

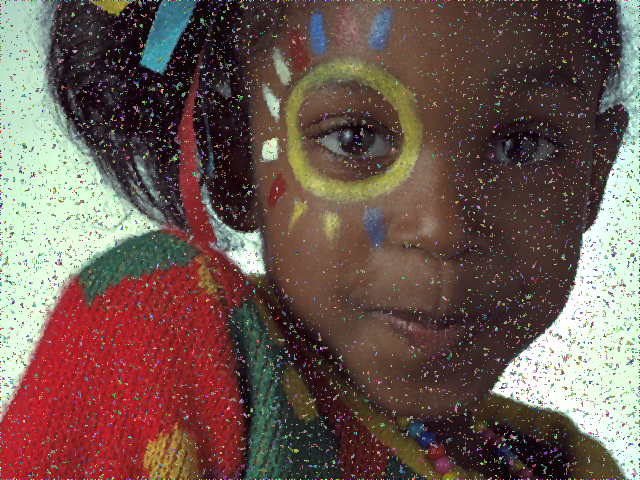

Supplement: Supplementary file 1 [file sensors-23-05657-s001.zip › sensors-2219351-supplementary/Sup_data/Exp_1_2/Restored/0.4/VMF/img03.png]

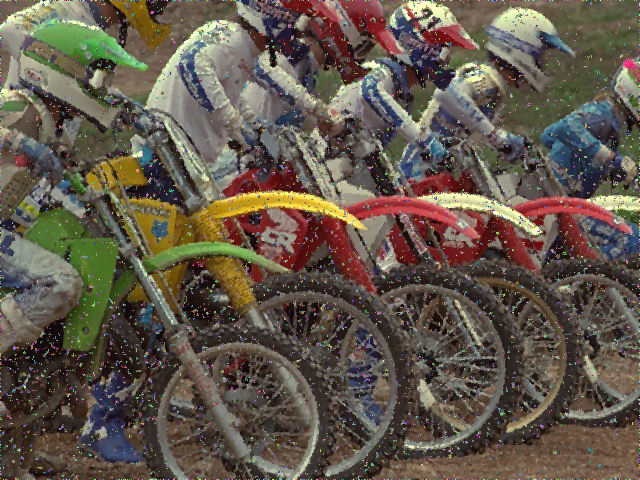

Supplement: Supplementary file 1 [file sensors-23-05657-s001.zip › sensors-2219351-supplementary/Sup_data/Exp_1_2/Restored/0.4/VMF/img04.png]

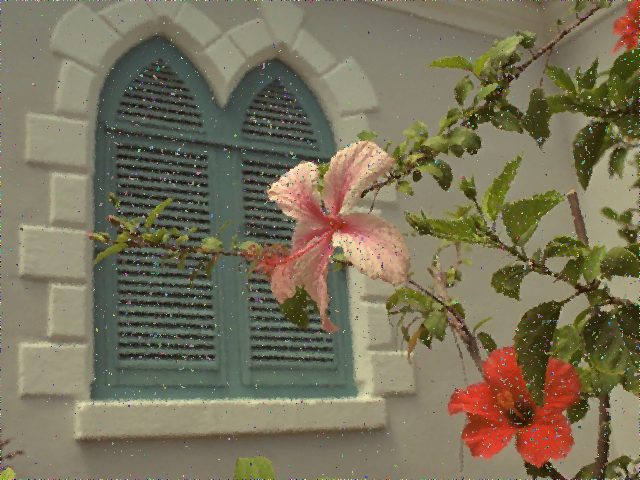

Supplement: Supplementary file 1 [file sensors-23-05657-s001.zip › sensors-2219351-supplementary/Sup_data/Exp_1_2/Restored/0.4/VMF/img05.png]

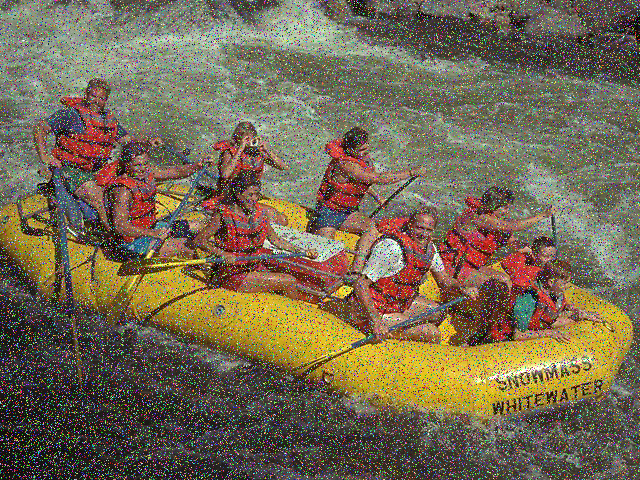

Supplement: Supplementary file 1 [file sensors-23-05657-s001.zip › sensors-2219351-supplementary/Sup_data/Exp_3/corImg.png]

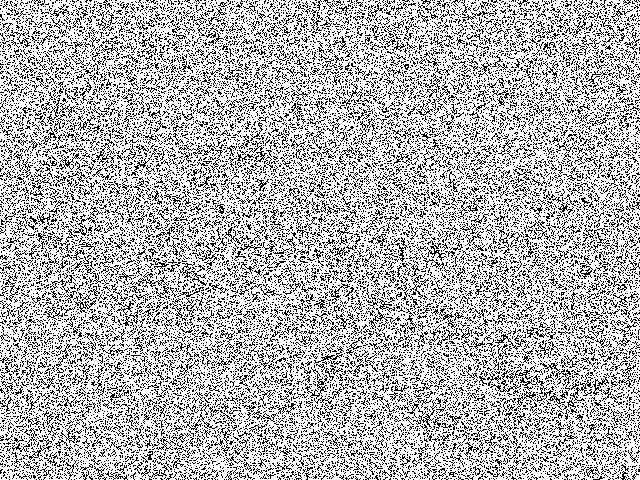

Supplement: Supplementary file 1 [file sensors-23-05657-s001.zip › sensors-2219351-supplementary/Sup_data/Exp_3/CWDM.png]

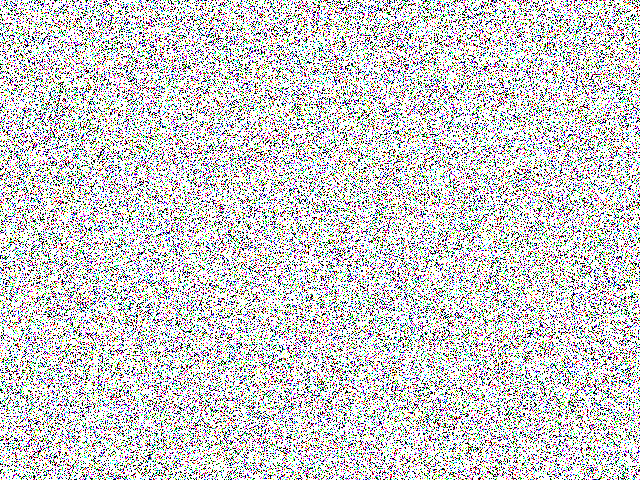

Supplement: Supplementary file 1 [file sensors-23-05657-s001.zip › sensors-2219351-supplementary/Sup_data/Exp_3/CWTM.png]

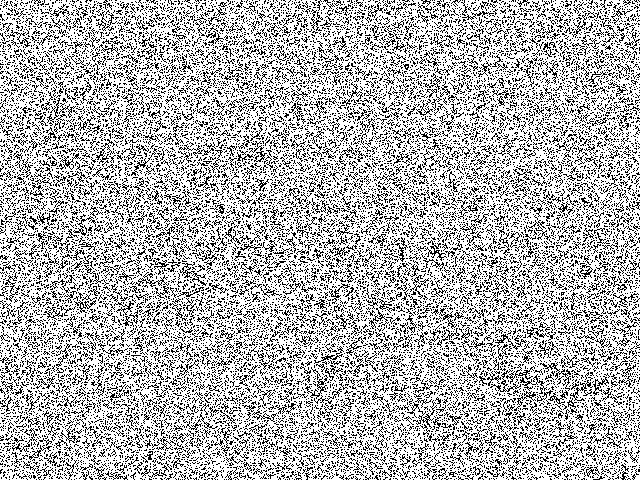

Supplement: Supplementary file 1 [file sensors-23-05657-s001.zip › sensors-2219351-supplementary/Sup_data/Exp_3/PWDM.png]

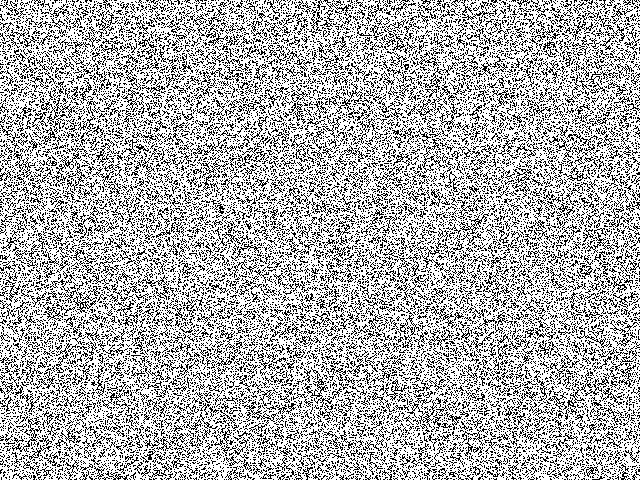

Supplement: Supplementary file 1 [file sensors-23-05657-s001.zip › sensors-2219351-supplementary/Sup_data/Exp_3/PWTM.png]

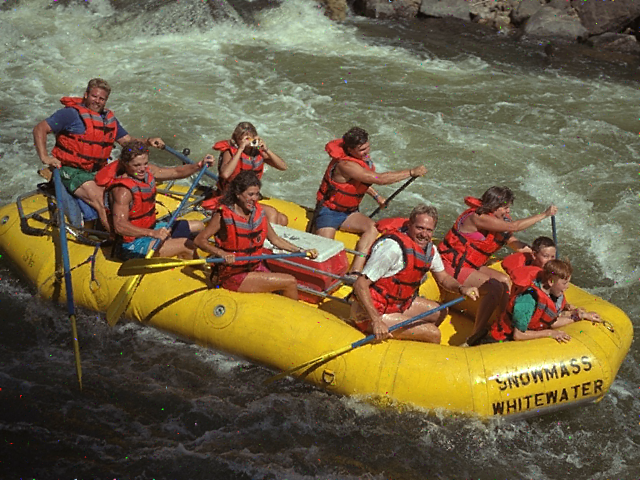

Supplement: Supplementary file 1 [file sensors-23-05657-s001.zip › sensors-2219351-supplementary/Sup_data/Exp_3/resImg.png]
